# Who Needs a Contractile Actomyosin Ring? The Plethora of Alternative Ways to Divide a Protozoan Parasite

**DOI:** 10.3389/fcimb.2019.00397

**Published:** 2019-11-21

**Authors:** Tansy C. Hammarton

**Affiliations:** Institute of Infection, Immunity and Inflammation, University of Glasgow, Glasgow, United Kingdom

**Keywords:** cell division, cytokinesis, protozoan parasite, actomyosin ring-independent cell division, budding, furrow ingression, abscission, cytofission

## Abstract

Cytokinesis, or the division of the cytoplasm, following the end of mitosis or meiosis, is accomplished in animal cells, fungi, and amoebae, by the constriction of an actomyosin contractile ring, comprising filamentous actin, myosin II, and associated proteins. However, despite this being the best-studied mode of cytokinesis, it is restricted to the Opisthokonta and Amoebozoa, since members of other evolutionary supergroups lack myosin II and must, therefore, employ different mechanisms. In particular, parasitic protozoa, many of which cause significant morbidity and mortality in humans and animals as well as considerable economic losses, employ a wide diversity of mechanisms to divide, few, if any, of which involve myosin II. In some cases, cell division is not only myosin II-independent, but actin-independent too. Mechanisms employed range from primitive mechanical cell rupture (cytofission), to motility- and/or microtubule remodeling-dependent mechanisms, to budding involving the constriction of divergent contractile rings, to hijacking host cell division machinery, with some species able to utilize multiple mechanisms. Here, I review current knowledge of cytokinesis mechanisms and their molecular control in mammalian-infective parasitic protozoa from the Excavata, Alveolata, and Amoebozoa supergroups, highlighting their often-underappreciated diversity and complexity. Billions of people and animals across the world are at risk from these pathogens, for which vaccines and/or optimal treatments are often not available. Exploiting the divergent cell division machinery in these parasites may provide new avenues for the treatment of protozoal disease.

## Introduction

Cytokinesis is the final stage of cell division, where the mother cell's cytoplasm, following the replication/duplication and segregation of cellular components, is partitioned, resulting in two daughter cells. To date, eukaryotic cell division has mostly been studied in cells of model organisms such as yeast, plants and animals. Cytokinesis is broadly considered to comprise the following key events (Pollard and Wu, 2010; Glotzer, 2017), although modifications occur during asymmetric division (Thieleke-Matos et al., 2017), insect embryogenesis (Xue and Sokac, 2016) and life cycle-related morphogenetic changes (Seiler and Justa-Schuch, 2010):

- division site selection (which often occurs much earlier in the cell cycle or even in the previous cell cycle).
- initiation signaling events.
- division machinery assembly.
- daughter cell partitioning by plasma membrane constriction (furrowing) or new membrane and cell wall construction (vesicle fusion).
- division machinery disassembly.
- daughter cell separation (abscission).

**Figure 1:**
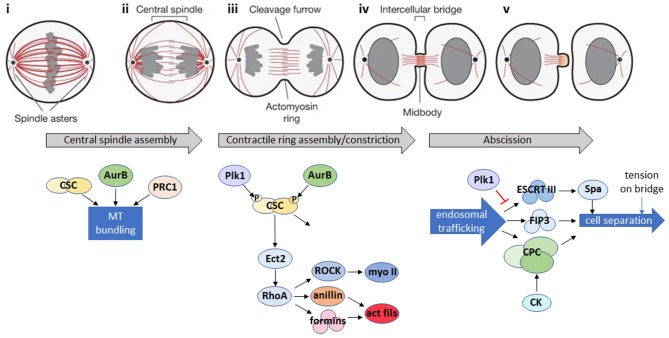
Animal cell cytokinesis. **Top:** schematic of the major events during cytokinesis in animal cells [gray: DNA; red: microtubules; adapted by permission from Springer Nature: ^©^(Fededa and Gerlich, 2012)]. **Bottom:** summary of the main signaling events during cytokinesis in animal cells. (i) During mitotic metaphase, condensed chromosomes align at the metaphase plate. (ii) Bipolar attachment of chromosomes to spindle microtubules releases the spindle attachment checkpoint and activates the anaphase promoting complex/cyclosome (APC/C), which degrades mitotic cyclin B and inactivates the mitotic cyclin-dependent kinase (CDK1). CDK1 inactivation triggers reorganization of the mitotic spindle into an array of antiparallel microtubule bundles (the central spindle) between the separating chromosomes. Microtubule bundling is promoted by Aurora B (AurB), the centralspindlin complex (CSC) and microtubule-bundling protein required for cytokinesis 1 (PRC1). (iii) A cortical contractile ring assembles from long formin-nucleated actin filaments and bipolar filaments of the motor, myosin II, and constricts to cleave the daughter cells. Actomyosin ring assembly is initiated in response to a signaling pathway where Polo-like kinase 1 (Plk1) and AurB phosphorylate the CSC, leading to activation of the Rho GDP-GTP exchange factor, Ect2, and its translocation to the cell cortex where it activates the RhoA GTPase. RhoA activates both myosin II (myo II) via the Rho kinase, ROCK, and formins which nucleate actin filaments (act fils), and recruits the scaffold protein anillin, resulting in the formation of actin and myosin filaments and subsequent assembly of the actomyosin ring. In addition to continued RhoA signaling, constriction of the actomyosin ring is influenced by changes in cortical tension, plasma membrane lipid composition at the site of furrow ingression, and by active force generation by the action of myosin motors (Emoto et al., 2005; Atilla-Gokcumen et al., 2014; Glotzer, 2017). (iv) The central spindle is compacted to form a microtubule-based midbody positioned in the center of a thin intercellular bridge that connects the daughter cells while the contractile ring is converted into a cortical midbody ring. (v) Endosomal trafficking of the Chromosomal Passenger Complex (CPC) and FIP3-endosomes, together with the Endosomal Sorting Complex Required for Transport III (ESCRT-III) filament system, which recruits the microtubule severing enzyme, spastin (Spa), act to remodel the intercellular bridge and bring about abscission, the final topological separation of the two daughter cells (Connell et al., 2009; Carmena et al., 2012; D'Avino and Capalbo, 2016). Additional regulators of abscission include citron kinase (CK), which works together with AurB in the CPC to stabilize the midbody architecture (Watanabe et al., 2013; McKenzie et al., 2016) and Plk1, which inhibits ESCRT-III recruitment to the midbody until late cytokinesis, when Plk1 is degraded (Bastos and Barr, 2010). Abscission is also regulated by tension on the intercellular bridge (Gould, 2016).

Within the evolutionary supergroups Opisthokonta (including metazoa and fungi) and Amoebozoa (amoebae), cytokinesis occurs via the assembly and constriction of a contractile actomyosin ring composed of filamentous actin, myosin II, and associated proteins during anaphase, perpendicular to the midpoint of the mitotic spindle (reviewed in Fededa and Gerlich, 2012; Pollard and O'Shaughnessy, 2019 and summarized in Figure 1). Members of all other supergroups (excepting perhaps *Naegleria* spp.) use different mechanisms to divide since they lack myosin II (Richards and Cavalier-Smith, 2005; Odronitz and Kollmar, 2007; Fritz-Laylin et al., 2010; Sebe-Pedros et al., 2014). Land plants and some green algae, for example, use vesicle delivery to assemble a phragmoplast composed of actin, microtubules, membranes and proteins, which partitions daughter cells (Livanos and Muller, 2019), while other green algae use a microtubule-based phycoplast (Cross and Umen, 2015). Parasitic protozoa use a plethora of alternative and divergent cytokinesis strategies.

### The Parasitic Protozoa and Myosin II

**Figure 2:**
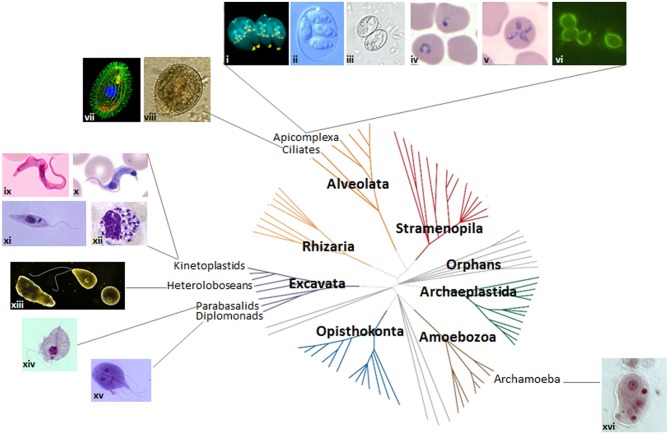
Protozoan parasites and the eukaryotic tree of life. Images of protozoan parasites (not to scale) mapped onto the seven domains of the eukaryotic tree of life. Adapted from Worden et al. (2015). Reprinted with permission from AAAS. (i) dividing *Toxoplasma gondii* (credit: Ke Hu and John M. Murray); (ii) *Eimeria maxima* (credit: S.J. Upton); (iii) *Sarcocystis* sporulated oocyst (credit: DPDx image gallery); (iv) *P. falciparum* ring stage (credit: DPDx image gallery); (v) *Theileria microti* (credit: DPDx image gallery); (vi) *Cryptosporidium parvum* (credit: EPA/H.D.A. Lindquist); (vii) *Tetrahymena thermophila* (credit: Dr. Muthugapatti Kandasamy, Director of the Biomedical Microscopy Core. University of Georgia bmc.uga.edu/); (viii) *Balantidium coli* (credit: Euthman); (ix) *Trypanosoma brucei* bloodstream stage (credit: CDC/Dr. Myron G. Schultz); (x) *T. cruzi* trypomastigote (credit: DPDx image gallery); (xi) *Leishmania* promastigote (credit: CDC/Dr. Mae Melvin/Public Health Image Library); (xii) *Leishmania* amastigotes in white blood cell (credit: DPDx image gallery); (xiii) *Naegleria fowleri* trophozoite, flagellated form and cyst (https://www.cdc.gov/parasites/naegleria/); (xiv) *Trichomonas* trophozoite (credit: DPDx image gallery); (xv) *Giardia* trophozoite (credit: schmidty4112), and (xvi) *Entamoeba histolytica* trophozoite (credit: Stefan Walkowski).

The protozoa are a diverse group of unicellular organisms, encompassing both free-living and parasitic species, with mammalian-infective species being found in the Amoebozoa (archamoebae), Excavata (heteroloboseans, parabasalids, diplomonads, and kinetoplastids), and Alveolata (ciliates and apicomplexans) supergroups (Figure 2). Of the myosin II-containing species, *Entamoeba* spp. are the best studied, yet there is no evidence for an actomyosin ring. Further, while it is possible that myosin II is key to cytokinesis in *Naegleria* spp., this has not yet been experimentally demonstrated. Most parasitic protozoa, though, lack myosin II, and some (*Giardia* and *Trichomonas*) lack functional myosins altogether (Richards and Cavalier-Smith, 2005; Odronitz and Kollmar, 2007; Sebe-Pedros et al., 2014), using e.g., alternative myosins or mechanisms relying on flagellar/ciliary motility and/or microtubule remodeling to divide. Further, some parasitic protozoa display relaxed cytokinesis regulatory mechanisms, with the apparent absence of canonical molecular checkpoints. Knowledge of the molecular regulation of cytokinesis is scant in some species, but substantial in others. Arguably, a greater knowledge of how these organisms divide is not just of interest from an evolutionary perspective given that model organisms are restricted to just three of the seven evolutionary supergroups (Figure 2) (Worden et al., 2015), but, given the significant morbidity and mortality caused by mammalian-infective parasitic protozoa, of great importance for developing novel parasite control strategies.

## Cytokinesis in the Amoebozoa

### *Entamoeba* spp. (Infraphylum Archamoebae) Divide by Cytofission

**Figure 3:**
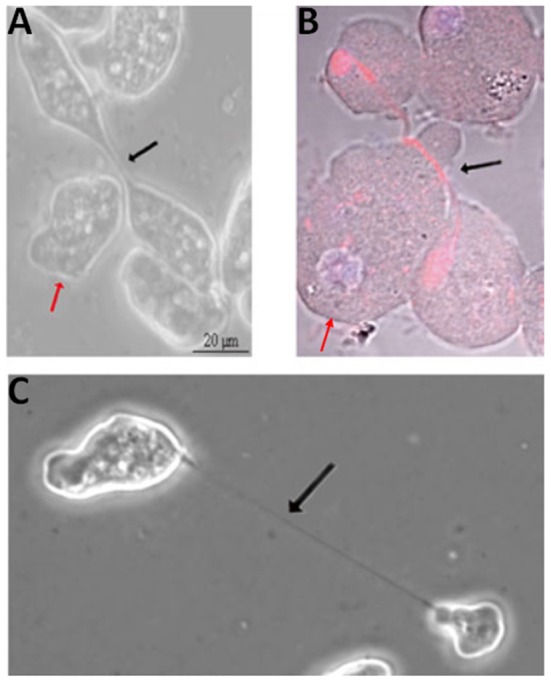
Cytokinesis in *Entamoeba histolytica*. Helper cells (red arrows) approaching **(A)** and migrating beneath (**B**; red: tubulin; blue: DNA) the intercellular bridge (black arrows) in dividing *E. histolytica* cells. **(C)** Dividing *E. histolytica* cells with an extended intercellular bridge. Adapted by permission from Springer Nature^©^ (Grewal and Lohia, 2015).

*Entamoeba* spp., which can cause dysentery and liver abscesses in humans and animals, display extremely plastic cell division. The *Entamoeba* genome is genetically heterogeneous, with individual trophozoites displaying ploidies of 1–10 *n* and varying numbers of nuclei. Cytokinesis involves plasma membrane constriction and formation/severing of an intercellular bridge (Figure 3), similar to other amoebae and animal cells. However, cell division is delinked from S phase and mitosis, occurring erratically and often asymmetrically, resulting in cells with no, one or multiple nuclei (Orozco et al., 1988; Lohia, 2003; Lohia et al., 2007; Mukherjee et al., 2009), with cell fusion events also contributing to multinuclear cell formation (Krishnan and Ghosh, 2018). Indeed, genome analysis suggests that *E. histolytica* encodes orthologs of all essential budding yeast cytokinesis proteins (although these have not been characterized), but that many cell cycle checkpoint proteins are not conserved (Grewal and Lohia, 2015), providing an explanation for the erratic division. Further, no actomyosin ring appears to be formed. Myosin II is present but has not yet been shown to convincingly localize to the constriction site, and actin forms longitudinal cables that run through the intercellular bridge (Majumder and Lohia, 2008). This is in contrast to the non-parasitic amoeba *Dictyostelium*, which does form a myosin-based contractile ring (De Lozanne and Spudich, 1987; Fukui, 1990; Fukui and Inoue, 1991), although since *Dictyostelium* cells lack a midbody, they utilize traction or external forces to complete abscission (Taira and Yumura, 2017). The *Entamoeba* intercellular bridge is severed mechanically as cells pull apart, although ~30% of cell cleavage events are aided by a helper or “midwife” cell migrating through the bridge, providing external mechanical force to ensure its scission (Biron et al., 2001; Mukherjee et al., 2009; Krishnan and Ghosh, 2018) (Figures 3A,B), as also observed in *Dictyostelium* (Tanaka et al., 2019). 10–20% of initiated cytokinetic events in *Entamoeba* are unsuccessful; the intercellular bridge retracts and daughter cells re-fuse.

Mechanical rupture or cytofission, uncoupled to the cell cycle, may be one of the earliest evolutionary mechanisms for cytokinesis, and is considered myosin II-independent, being also employed by *Dictyostelium* cells lacking myosin II (Taira and Yumura, 2017). However, the myosin II inhibitor, 2,3-butanedione monoxime, inhibits cytofission in *E. invadens* multinuclear giant cells, suggesting that at least in these cells, cytofission may be dependent on myosin II (Krishnan and Ghosh, 2018). Other likely *Entamoeba* cytokinesis proteins include actin, Ehformin-1 and Ehformin-2, which localize to the constriction site and the intercellular bridge, along with tubulin and the calcium- and β-tubulin-binding protein, EhCaBP6 (Chavez-Munguia et al., 2006; Majumder and Lohia, 2008; Grewal et al., 2013; Verma et al., 2017). Overexpression of Ehformin-1 [whose activity is regulated by Rho1 GTPase (Bosch et al., 2012)] or Ehformin-2 increases ploidy and formation of binucleate/multinucleate cells (Majumder and Lohia, 2008). Further, overexpression of the kinase domain of p21-activated kinase, EhPAK2 (Arias-Romero et al., 2006), or constitutive activation of EhRACA (Ghosh and Samuelson, 1997) or EhRACG (Guillen et al., 1998) also inhibit cytokinesis in ~10% cells, leading to the formation of multinucleate cells. The EhPC4 transcription factor seems to regulate cytokinesis (in addition to DNA replication), with its overexpression upregulating expression of various cell cycle proteins, e.g., EhNUDC, a nuclear movement protein ortholog, inhibiting cytokinesis and resulting in the formation of multinucleate cells (Hernández De La Cruz et al., 2016). Finally, analysis of the *Entamoeba* protein kinome suggests that some canonical regulators are present, but unusual and divergent signaling pathways may also operate (Anamika et al., 2008).

## Cytokinesis in the Excavata

Parasitic Excavata exhibit a wide variety of division mechanisms. While *Naegleria* potentially divides via a conventional actomyosin ring, the majority of excavate parasites divide along their long axis, employing mechanisms that heavily rely on flagellar motility and cytoskeletal rearrangements, and are regulated by complex signaling pathways.

### *Naegleria* spp. (Class Heterolobosea): Myosin II Is Present, but Is It Required for Cytokinesis?

Despite not being an Amoebozoa, *Naegleria* reproduces as an amoeboid form, although it also forms a transient, non-dividing flagellated form and a cyst form. Most *Naegleria* spp. are free-living protists that feed on bacteria. However, *N. fowleri* is an opportunistic human pathogen, causing usually fatal primary amoebic meningoencephalitis. Little attention has been paid to *Naegleria* cytokinesis, despite detailed studies of nuclear division having revealed unusual and divergent features (Schuster, 1975; Gonzalez-Robles et al., 2009; Walsh, 2012). However, dividing cells appear to constrict at the division site with daughter cells linked by an intercellular bridge prior to abscission, similar to animal cells, although it is not known if an actomyosin ring forms (Gonzalez-Robles et al., 2009; Walsh, 2012). *N. gruberi* genome analysis has revealed the presence of genes encoding myosin II as well as complete actin and microtubule cytoskeletons, although in the amoeboid form, microtubules are only present within the mitotic spindle (Fulton and Simpson, 1976; Chung et al., 2002; Fritz-Laylin et al., 2010, 2011). However, the signaling networks regulating cell division remain to be elucidated, and given that the genome encodes at least 265 protein kinases, 32 protein phosphatases and 182 Ras GTPases, along with 21 RhoGEFs and 25 RhoGAPs, but apparently no Rho GTPase genes, they are likely to be complex and potentially divergent (Fritz-Laylin et al., 2010, 2011).

### *Giardia* (Order Diplomonadida): Flagellar Motility, Vesicle Trafficking, and Cytoskeletal Rearrangements Are Key

**Figure 4:**
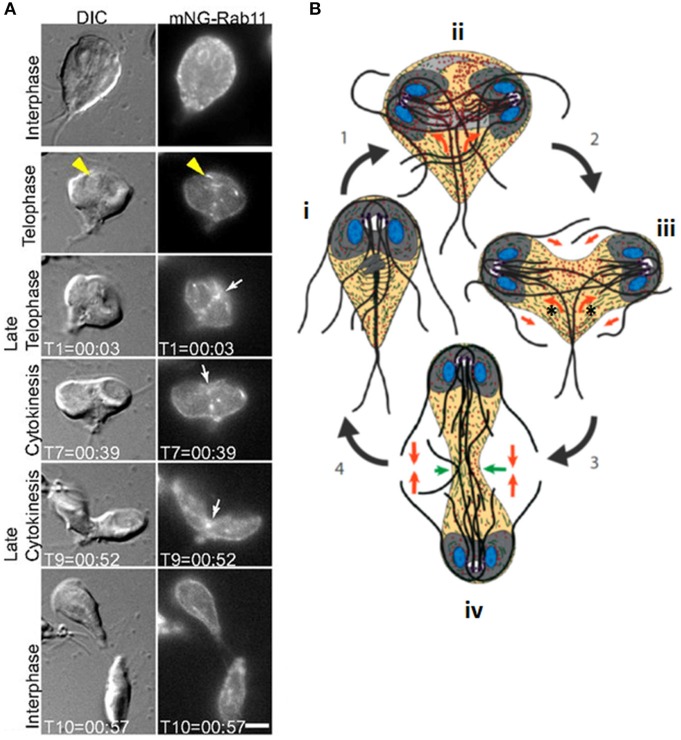
Cytokinesis in *Giardia*. **(A)** Stills from time-lapse imaging of an mNeonGreen-Rab11-expressing *Giardia* trophozoite undergoing cytokinesis. Rab11 is recruited to the intracytoplasmic flagellar axonemes (yellow arrowheads) in telophase and marks the cleavage furrow just prior to and during furrow ingression (white arrows). Scale bar: 5 μm. **(B)**: cartoon of dividing *Giardia* trophozoite. Nuclei are shown in blue, ventral discs in gray and flagella as dark gray/black lines. An interphase cell (i) initiates mitosis (1). Actin (green) positions the microtubule cytoskeleton (ii). Rab11 (red) labels the intracytoplasmic axonemes; newly-forming axonemes guide Rab11 to the furrow (orange arrows). Furrow ingression (2) is initiated in response to the thinning of overlapping microtubules in the parental ventral disc (light gray), which allows it to open to a C-shaped conformation (new daughter cell ventral discs are in dark gray), and opposing forces (orange arrow) from the intracytoplasmic caudal axonemes (asterisks) (iii). (3) Caudal flagella flexion continues to drive the daughter cells apart and reduced cortical actin at the leading edge of the furrow (green arrows, iv) ensures continued furrow ingression. (4) Flagellar motility and Rab11-mediated trafficking of membrane remodeling proteins results in abscission. Adapted from Hardin et al. (2017).

*Giardia*, also known as *Giardia lamblia, G. intestinalis*, or *G. duodenalis*, is a waterborne diplomonad that causes the diarrhoeal disease, Giardiasis. It exists as a proliferative flagellated trophozoite and a water-resistant cyst. Trophozoites have two genetically identical diploid nuclei, and four pairs of flagella (anterior, posterolateral, ventral and caudal) (McInally and Dawson, 2016). There are also several other microtubule-based structures including the ventral disc, which mediates surface attachment, and the median body, which lies perpendicular to the caudal axonemes and is present only up until mitosis (Hardin et al., 2017). Cytokinesis proceeds along the long axis of the cell, from the anterior end (Figure 4), and is promoted by remodeling of the parental ventral disc following mitotic spindle disassembly (Hardin et al., 2017). Daughter cells move in opposite directions, receiving two nuclei derived from each of the mother cell nuclei (Sagolla et al., 2006).

Flagellar forces appear to be critical for *Giardia* cytokinesis. Inhibiting flagellar motility via depletion of axonemal central pair protein, PF16, results in a 10-fold increase in cells with four or more nuclei, leading to the proposal that furrowing initiates in response to sustained bending of the caudal flagella, while anterior flagella motility may contribute to abscission (Hardin et al., 2017). This flagellar motility-based division is highly efficient, with mitosis occurring in ~6.5 min, and cytokinesis taking just 50 s, some 30–90 times faster than has been reported in plants, fungi, and mammalian cells. Cytokinesis does not proceed uniformly; furrow ingression occurs at a faster rate than abscission, suggesting sequential action of cell division components. Further, Brefeldin A treatment (which disrupts trafficking from the endoplasmic reticulum) arrests furrowing at a defined point, approximately midway through ingression, indicating that additional membrane/proteins are required to complete cytokinesis (Hardin et al., 2017). Cells that arrest partway through cytokinesis are heart-shaped, and although heart-shaped cells were formerly thought to be a normal cytokinesis stage (Benchimol, 2004a; Sagolla et al., 2006; Paredez et al., 2011), it has recently been proposed that such cells are abnormal cells that are not actively dividing (Hardin et al., 2017).

Actin is also key for *Giardia* cytokinesis, since its knockdown stalls furrow ingression and delays abscission or blocks cytokinesis completely in a subset of cells. However, actin does not mark the division furrow, and is instead enriched around spindles and developing axonemes, likely helping to position these structures (Paredez et al., 2011). Actin abundance is reduced just ahead of the leading edge of the furrow, which may promote furrow ingression via alterations in cortical tension, as in other organisms (Hardin et al., 2017). Actin is also required for protein trafficking (Paredez et al., 2011) potentially supporting vesicular trafficking required for furrowing/abscission. The GTPase, Rab11, is also key for vesicle trafficking and cytokinesis, and its knockdown extends or prevents cytokinesis (Hardin et al., 2017). Rab11 delineates the cleavage furrow during pre-furrowing (where the cell membrane invaginates, but has not yet cleaved), remaining in the furrow and marking its leading edge during cytokinesis (Figure 4). Further, it marks intracytoplasmic flagellar axonemes late in telophase and the plus ends of growing axonemes, co-localizing with actin, and it has been proposed that developing axonemes are positioned to perform a midbody/phragmoplast-like role in directing trafficking to the furrow (Hardin et al., 2017). Sphingolipid biosynthesis also appears to be important for *Giardia* cytokinesis since treatment with the glucosylceramide synthase (GCS) inhibitor, DL-*threo*-1-phenyl-2-palmitoylamino-3-morpholino-1-propanol (PPMP), or increasing ceramide levels, significantly inhibited cytokinesis leading to the appearance of large proportions of doublet (heart-shaped) and triplet cells (Sonda et al., 2008; Stefanic et al., 2010). Further, GCS appears to be important for vesicular trafficking. Another sphingolipid, psychosine, has also been reported to inhibit *Giardia* cytokinesis (Stefanic et al., 2010).

Several canonical mitotic and/or cytokinesis regulators, including CDK1-like kinases, a Wee1 kinase, an Aurora kinase (AK), and a Polo-like kinase (PLK) are present in *Giardia* (Manning et al., 2011), although cell cycle protein degradation machinery (APC and SCF complexes) is missing (Gourguechon et al., 2013). Differential expression profiles of cell cycle proteins across the cell cycle have been mapped (Horlock-Roberts et al., 2017), but their cell cycle-dependent degradation is not always mediated via the ubiquitin pathway (Gourguechon et al., 2013). Of the putative cell cycle kinases, only AK has been studied functionally. AK inhibitors block completion of *Giardia* cytokinesis (Davids et al., 2008). AK co-localizes with its potential substrate, microtubule end-binding protein 1 homolog, GlEB1, at the nuclear membrane and median body during interphase, and at the mitotic spindle during mitosis. GlEB1 is phosphorylated on S148 by AK *in vitro*, a phosphosite that, when mutated *in vivo*, leads to defects in cytokinesis (Kim et al., 2017), suggesting AK plays roles in the disassembly/reorganization of the cytoskeleton during mitosis and cytokinesis. Further, GlEB1 also interacts with Glγ-tubulin *in vivo*; depletion of Glγ-tubulin or two γ-tubulin complex proteins, GlGCP2, and GlGCP3, results in cytoskeletal defects, including defects in median body formation and flagellar structural defects, and inhibition of furrowing (Kim and Park, 2019).

### *Trichomonas* spp. (Parabasalia): Flagellar Motility and Cytoskeletal Rearrangements Are Important

**Figure 5:**
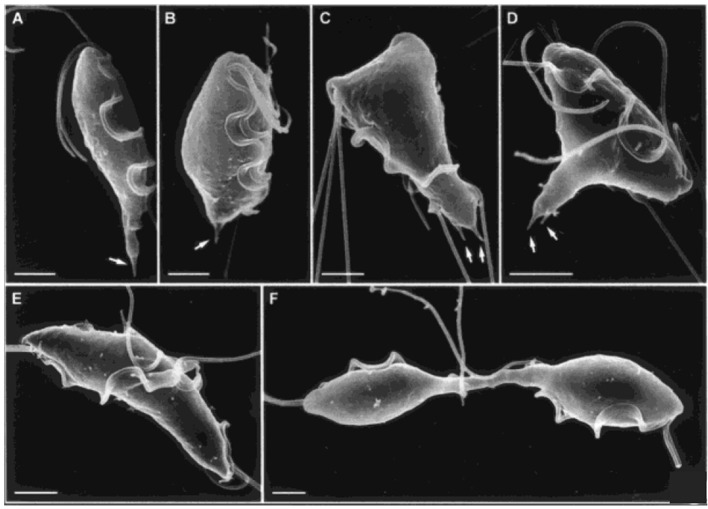
Cytokinesis in *Trichomonas*. Scanning electron microscopy images of *T. fetus* at different cell cycle stages. **(A)** interphase; **(B)** premitosis; **(C)** prophase; **(D)** metaphase; **(E)** anaphase; **(F)** telophase with daughter cells connected by an axostylar trunk. Scale bars: 3 μm **(A,F)**; 2 μm **(B–E)**. Arrows: axostyle tips. Adapted by permission from Cambridge University Press^©^ Microscopy Society of America (Benchimol, 2004b).

Trichomonads are flagellated protists that cause trichomoniasis in some hosts; the human pathogen, *Trichomonas vaginalis*, which infects ~3% of the world's population annually (Kusdian et al., 2013), and the cattle and feline pathogen, *Tritrichomonas fetus*, are among the best studied. In culture, trichomonads have a tear-drop shape during interphase, but transform to multinucleate amoeboid forms when in contact with epithelial cells, and will form pseudocysts under stress (Benchimol, 2004b). Like *Giardia*, they have a complex cytoskeleton, comprising various microtubule (axostyle, pelta, basal bodies, flagella, and mitotic spindle) and proteinaceous (costa, rootlet, and parabasal filaments) structures (Benchimol, 2004b; Preisner et al., 2016). Actin is also present throughout the cell (Brugerolle et al., 1996; Kusdian et al., 2013). During cell division, basal body migration, signaling the start of mitosis, and flagellar-driven propulsion lead to morphological changes that drive crossing of duplicated axostyles, resulting in constriction of the nucleus during karyokinesis and thinning of the connection between the daughter cells, such that by telophase, daughter cells remain connected only by an axostylar trunk (Ribeiro et al., 2000) (Figure 5). Flagellar motility then rotates the daughters around their axis, likely providing torsional forces to help drive cytokinesis to completion (Ribeiro et al., 2000; Benchimol, 2004b). However, despite detailed ultrastructural studies of mitosis and cytokinesis, very little is known of their molecular regulation (Amador et al., 2017).

### *Trypanosoma* and *Leishmania* spp. (Class Kinetoplastea): Complex Signaling, Microtubule Cytoskeleton Remodeling, and Flagellar Motility Regulate Longitudinal Furrow Ingression

The defining feature of the Kinetoplastea is the kinetoplast, a disc-shaped structure containing the DNA of the single mitochondrion (kDNA), although some *Trypanosoma brucei* subspecies e.g., *T. b. evansi* and *T. b. equiperdum* have lost all or part of their kDNA (Lai et al., 2008). Some kinetoplastids are free-living; others infect animals and plants, often being transmitted between hosts by insect vectors. Among the best studied are the mammalian pathogens, *Trypanosoma brucei* spp. (and to a lesser extent, *T. congolense*), the causative agents of human and/or animal African trypanosomiasis (HAT/AAT), *Trypanosoma cruzi*, the causative agent of Chagas disease, and *Leishmania* spp., which cause the Leishmaniases. Molecular studies of cytokinesis have mainly been confined to *T. b. brucei* due to the availability of molecular genetic tools. While there are undoubtedly many similarities between different kinetoplastids, a variety of structural and developmental differences will likely influence cell division and its molecular control. Recent tool development (Duncan et al., 2016; Beneke et al., 2017; Gibson et al., 2017; Costa et al., 2018; Lander et al., 2019) should accelerate progress and shed light on these differences.

#### *T. brucei* and Other Animal Trypanosomes

**Figure 6:**
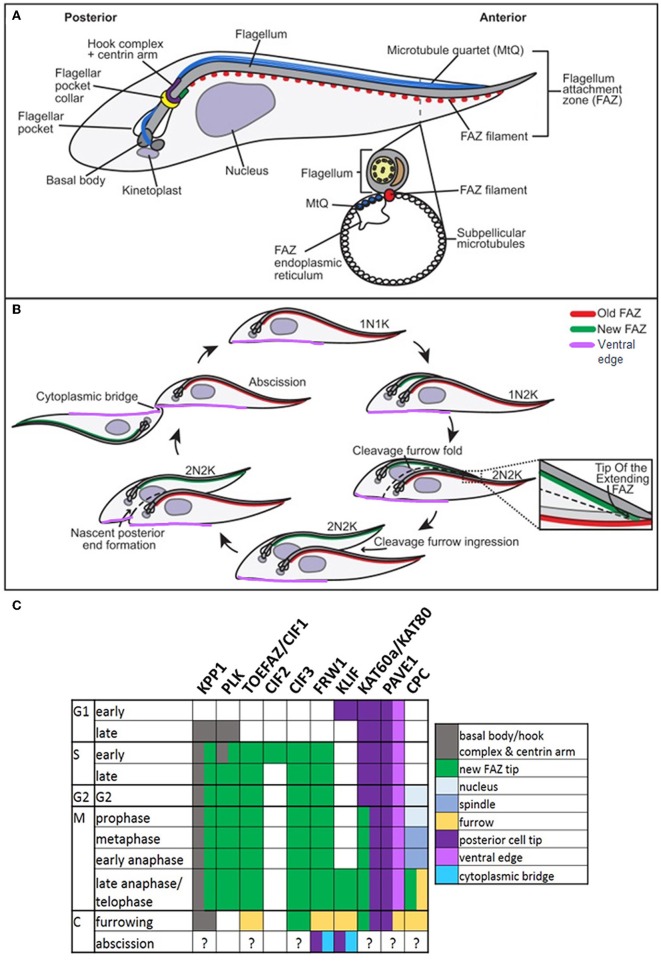
Cytokinesis in *Trypanosoma brucei*. Cartoons of cytoskeletal structures **(A)** and cell division **(B)** in procyclic *T. brucei*. N: nucleus; K: kinetoplast. Adapted with permission from Journal of Cell Science (Sinclair-Davis et al., 2017). **(C)** Table indicating the cellular localizations of key cytokinesis proteins. KPP1 and PLK co-localize throughout the cell cycle from late G1, until late anaphase, when PLK becomes undetectable. TOEFAZ1/CIF1 localizes to the Tip Of the Extending FAZ (or new FAZ tip) (highlighted in **B**), and acts as a scaffold for many other cytokinesis proteins, including KPP1, PLK1, CIF2, CIF3, FRW1, KLIF, KAT60a/KAT80, and PAVE1. The location of PAVE1 at the ventral edge of the old-flagellum daughter is indicated in purple in **(B)**. The localization of the chromosomal passenger complex (CPC) is also indicated **(C)**. Note that the localizations of many of these proteins have not been specifically determined at abscission.

**Figure 7:**
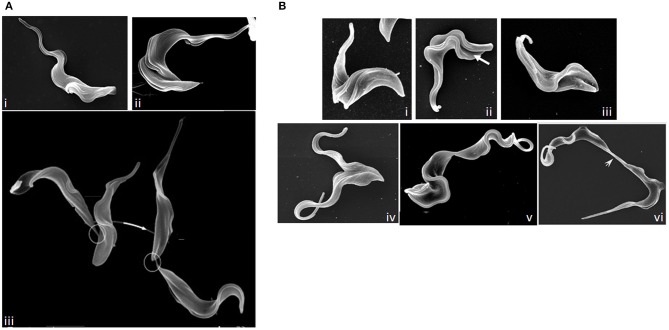
Images of dividing *T. brucei*. **(A)** Scanning electron microscopy (SEM) images of procyclic *T. brucei* undergoing division. (i) cell with two flagella but no furrow (Gull Lab of courtesy of Sue Vaughan); (ii) cell with a cleavage fold (Gull Lab courtesy of Catarina Gadelha), and (iii) cells undergoing abscission (adapted from Wheeler et al., 2013). Note the differing posterior end morphologies of the daughter cells and the migration of the intercellular bridge (circled) as cells progress through abscission. **(B)** SEM images of dividing bloodstream form *T. brucei*. (i) cell with two flagella but no furrow; (ii) formation of a cleavage fold (arrow); (iii) remodeling of the posterior ends; (iv) cleavage furrow ingression; (v) abscission; and (vi) late abscission with an extended intercellular bridge (arrowhead). Note that the intercellular bridge is thicker than in procyclic cells and does not migrate. Images (i–v) Tansy Hammarton; image (vi) adapted from Zhang et al. (2019a).

*T. brucei, T. congolense*, and *T. vivax* have complex life cycles split between mammalian and tsetse fly hosts, comprising a series of morphologically and metabolically distinct stages. These may be replicative (bloodstream trypomastigotes in the mammal, and procyclic and epimastigote forms in the fly) or cell cycle arrested (Van Den Abbeele et al., 1999; Matthews, 2005; Gluenz et al., 2008; Rotureau et al., 2012; Capewell et al., 2016; Trindade et al., 2016; Peacock et al., 2018). Cell division has been best studied in *T. brucei* in the procyclic form; more limited studies in bloodstream and epimastigote forms have highlighted similarities alongside key structural/molecular differences. *T. brucei* is vermiform and flagellated; a corset of parallel subpellicular microtubules confer a helical axis and polarity (Hemphill et al., 1991; Robinson et al., 1995; Gull, 1999), while a single motile flagellum emerges from a flagellar pocket at the posterior of the cell and is linked laterally to the cell body by a flagellum attachment zone (FAZ) (Figure 6A) (Gull, 1999). Following organelle replication, the cytoskeleton is remodeled locally in the vicinity of the furrow site in preparation for cytokinesis, but does not globally break down (Sherwin and Gull, 1989). New subpellicular microtubules are inserted in between the duplicated FAZ microtubule quartets (Wheeler et al., 2013). The plasma membrane then invaginates between the duplicated flagella, forming a cleavage fold (Figures 6B, 7) and microtubules are remodeled at the posterior end to create nascent daughter cell tips (Wheeler et al., 2013). The cleavage furrow/cleft then initiates from the anterior end of the new FAZ (Robinson et al., 1995; Kohl et al., 2003; Zhou et al., 2011) and ingresses posteriorly (Figures 6B, 7). Significant molecular evidence (see below) suggests that there are at least two distinct stages in furrow ingression: separation of the cell bodies up to roughly the midpoint between the segregated daughter cell nuclei, and then continued ingression to cleave the nascent posterior ends. In the procyclic, but not bloodstream form, a flagellar connector holds daughter flagella together at their anterior tips until cleavage furrow/cleft formation is complete (Moreira-Leite et al., 2001; Wheeler et al., 2013). Just before abscission, the flagellar ends dissociate, allowing the cell bodies to move apart, now connected by just a thin cytoplasmic bridge containing microtubules (Figures 6B, 7A). This initially links the posterior tip of the old-flagellum daughter with the side of the new-flagellum daughter, before migrating to connect the daughter posterior tips (Figure 7A). The end of cytokinesis is morphologically distinct in the bloodstream form (Figure 7B); the cytoplasmic bridge is thicker and links the cells only at their posterior tips. In both life cycle stages, it takes a while to resolve the cytoplasmic bridge, and bloodstream form cells may undergo a second cell cycle before abscission is completed (Wheeler et al., 2013).

**Figure 8:**
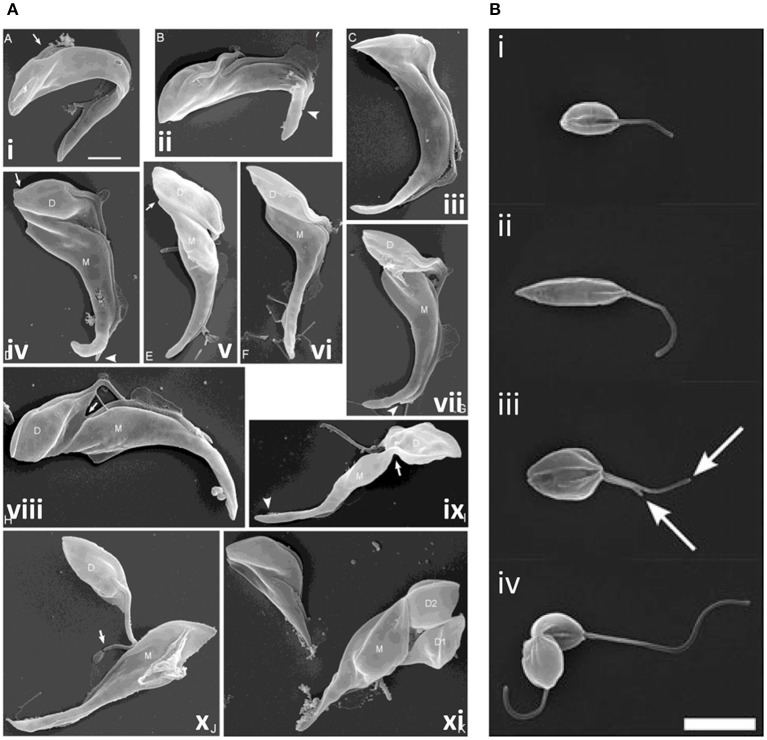
Cytokinesis in *Trypanosoma congolense* and *Leishmania mexicana*. **(A)** Scanning electron microscopy (SEM) images of *T. congolense* proventricular cells undergoing cell division (adapted from Peacock et al., 2018). (i) emergence of the new flagellum (arrow); (ii) extension of the new flagellum, but not to the tip of the old flagellum (arrowhead); (iii) cleavage fold formation; (iv–vii) cleavage furrow ingression and formation of nascent ends of mother (M) and daughter (D) cells (arrows). Arrowheads indicate distal tip of old flagellum. (viii) late-stage cleavage furrow ingression (arrow), pre-abscission; (ix) formation of cytoplasmic bridge (arrow); (x) completion of abscission; (xi) mother cell (M; left) with two sequentially-produced daughter cells, D1 and D2. Scale bar = 2 μm. **(B)** SEM images of dividing *L. mexicana* cells (taken from Wheeler et al., 2011). (i) Cell in early G1 phase with one flagellum; (ii) elongated cell (in later G1/ S phase); (iii) mitotic cell with two flagella (arrows); and (iv) cell in abscission. Scale bar: 5 μm.

Additional cytokinesis events occur during epimastigote development in the tsetse fly; *T. brucei* proventricular cells undergo an asymmetric division to produce long and short epimastigote daughters (Van Den Abbeele et al., 1999); in contrast, *T. congolense* remodels to form epimastigotes (Peacock et al., 2018). Short epimastigotes can divide to produce two epimastigote daughters [*T. brucei, T. congolense, T. vivax*, and *T. lewisi* (a rat trypanosome transmitted by fleas)], divide asymmetrically to give epimastigote and pre-metacyclic trypomastigote daughters (*T. brucei, T. vivax*, and *T. congolense*) or interconvert to trypomastigotes (*T. lewisi*) (Van Den Abbeele et al., 1999; Gluenz et al., 2008; Peacock et al., 2012, 2018; Rotureau et al., 2012; Ooi et al., 2016; Zhang et al., 2019a,c). While epimastigote furrow ingression appears to progress from anterior to posterior in all species, as in bloodstream and procyclic *T. brucei*, there are some notable differences in abscission. In *T. congolense* (Figure 8A), daughter cells are rarely joined tip to tip; instead, the anterior of the new-flagellum daughter is attached to the side of the old-flagellum daughter (termed the “mother” cell in Peacock et al., 2018). Further, the mother cell rapidly divides again, while still attached to its first daughter, resulting in a rosette of three cells; daughter cell motility is thought to ensure final severing of the link to the mother cell (Peacock et al., 2018). *T. lewisi* epimastigotes also undergo several rounds of cell division without completing cytokinesis, rapidly forming rosettes with 5–6 cell bodies attached at their posterior ends, from which, periodically, a fully segmented cell is released (Zhang et al., 2019c).

Cytokinesis in *T. brucei* requires the prior duplication and segregation of various single copy organelles and structures, with FAZ duplication being critically important (Hammarton et al., 2003; Kohl et al., 2003; Chanez et al., 2006; Hall et al., 2006; Rodgers et al., 2007; Li and Wang, 2008; Barquilla and Navarro, 2009; Zhou et al., 2010). Further, flagellar motility (Branche et al., 2006; Broadhead et al., 2006; Ralston et al., 2006) and plasma membrane composition (Lillico et al., 2003; Sheader et al., 2005; Rodgers et al., 2007; Fridberg et al., 2008; Schoijet et al., 2018) are also key. Molecular regulation of cytokinesis has probably been more extensively investigated in *T. b. brucei* (Figure 6C) than in any other protozoan parasite, although studies have been restricted to *in vitro*-cultured procyclic and bloodstream forms, despite *in vitro* differentiation of procyclic parasites to epimastigote and metacyclic forms being possible (Kolev et al., 2012). Some significant molecular differences between the life cycle stages have been revealed, including different checkpoints. For example, inhibiting mitosis blocks cytokinesis in the bloodstream form, but not the procyclic form (Hammarton et al., 2003). Further, the anaphase-promoting complex/cyclosome (APC/C) appears to promote the metaphase to anaphase transition in procyclic cells, as in other eukaryotes, but the anaphase to telophase transition in bloodstream form cells (Kumar and Wang, 2005; Bessat et al., 2013; Bessat, 2014).

Additionally, while many canonical cell cycle regulators are present in *T. brucei*, their function may be divergent and they often act together with trypanosome-specific regulators. For example, the trypanosome ortholog of the multifunctional protein kinase and key mitotic regulator, polo-like kinase (TbPLK), appears to be non-essential for mitosis. Instead, it plays critical roles in basal body segregation, flagellum biogenesis, attachment and segregation, and cytokinesis (Kumar and Wang, 2006; Hammarton et al., 2007; de Graffenried et al., 2008, 2013; Li et al., 2010; Ikeda and de Graffenried, 2012; Lozano-Nunez et al., 2013). Interestingly, several kinetochore proteins contain a divergent polo-box domain suggesting that an ancestral PLK may have duplicated and functionally diverged (Nerusheva and Akiyoshi, 2016). Further, while the SCF (Skp1-Cdc53/Cullin-F-box) protein degradation complex regulates the G1/S transition in metazoans, the trypanosome F-box proteins CFB1 and CFB2, along with TbCDC34, a homolog of the E2 ubiquitin-conjugating enzyme usually associated with the SCF complex, modulate progression through bloodstream form cytokinesis (Benz and Clayton, 2007; Rojas et al., 2017). Additionally, while canonical protein kinase A (or cAMP-dependent kinase) regulates a multitude of signaling pathways in response to cAMP, TbPKA is not activated by cyclic nucleotides and is essential for cytokinesis, potentially via phosphorylation of flagellar and cytoskeletal targets and/or cell cycle proteins such as mitotic cyclin 6, the Nuclear-DBF2-related (NDR) kinase, TbPK50 and TOEFAZ1/CIF1 (see below) (Jones et al., 2014; Bachmaier et al., 2019).

The aurora kinase, TbAUK1, is vital for mitosis and progression into cytokinesis in procyclic and bloodstream *T. brucei* (Li and Wang, 2006; Tu et al., 2006; Li et al., 2007, 2009) as in other eukaryotes, but acts alongside two kinetoplastid-specific proteins, CPC1 and CPC2, forming a divergent chromosomal passenger complex (CPC) (Li et al., 2008a). The chromosomal passenger complex assembles at the spindle midzone together with two novel nuclear kinesins (TbKINA and TbKINB) and the tousled like kinase, TbTLK1 (Li et al., 2008a,b), translocating at late anaphase/telophase to the anterior tip of the new FAZ before traveling with the leading edge of the ingressing furrow toward the posterior cell tip (Li et al., 2008a, 2009). In the procyclic form, recruitment of the chromosomal passenger complex to the new FAZ tip relies on the scaffold protein TOEFAZ1/CIF1 (tip of extending FAZ protein 1/cytokinesis initiation factor 1). TOEFAZ1/CIF1 first appears (after being phosphorylated by TbPLK at the centrin arm or hook complex) at the new FAZ tip early in S phase, remaining there until cytokinesis, when it tracks the leading edge of the ingressing furrow (Zhou et al., 2016a). TbBOH1 (bait on hook protein 1) is required for TOEFAZ/CIF1 and PLK localization to the new FAZ tip (Pham et al., 2019). Further, TOEFAZ1/CIF1 also requires the FAZ tip localizing Protein Required for Cytokinesis, FPRC, to localize to the new FAZ tip and the cleavage furrow. Interestingly, FPRC is also found at the old FAZ tip and, in turn, requires the cytokinesis initiation factor, CIF4, to localize to the FAZ tips and the furrow (Hu et al., 2019). TOEFAZ1/CIF1 keeps TbPLK at the new FAZ tip from S phase to anaphase (Mcallaster et al., 2015; Zhou et al., 2016a), recruiting TbAUK1 to the new FAZ tip at late anaphase when TbPLK is lost from this site (Zhou et al., 2016a). Interactions with two other cytokinesis initiation factors, CIF2 and CIF3, are also important for maintaining TOEFAZ1/CIF1 localization at the new FAZ tip and for TbAUK1 recruitment (Zhou et al., 2016b; Kurasawa et al., 2018). In bloodstream form trypanosomes, CIF1-3 display similar localizations to those in the procyclic form, but their interactions with TbAUK1 and TbPLK, and the localizations of FPRC and CIF4 have not yet been investigated (Zhang et al., 2019a). Depletion of any of the CIF proteins in procyclic and/or bloodstream *T. brucei* affects furrow ingression, impairing its accuracy or blocking normal furrowing completely (Zhou et al., 2016a,b; Sinclair-Davis et al., 2017; Kurasawa et al., 2018; Hu et al., 2019; Zhang et al., 2019a). In procyclic, but not bloodstream form trypanosomes, CIF1, CIF2, CIF4, or FPRC depletion has been reported to result in furrow ingression occurring in the opposite direction, from the posterior to the anterior, which was interpreted as indicating a backup cytokinesis pathway was turned on (Zhou et al., 2016a,b; Hu et al., 2019; Zhang et al., 2019a). However, why such a pathway should operate, and in only one life cycle stage, is unclear. An alternative explanation is that the “posterior-anterior furrowing” instead reflects continued normal nascent daughter cell posterior end remodeling (Wheeler et al., 2013) in the absence of furrow ingression, as previously observed following FAZ10 or katanin depletion (Benz et al., 2012; Moreira et al., 2017), with continued flagellar motility acting to pull apart the nascent posterior ends (Sinclair-Davis et al., 2017). Intriguingly, one further difference between bloodstream and procyclic trypanosomes, is that a flagellar axonemal inner-arm dynein complex, comprising dynein heavy chain ortholog TbIAD5-1 and TbCentrin3,is essential for CIF1-3 localization, and therefore, for cytokinesis in the bloodstream form, but not in the procyclic form (Zhang et al., 2019b). However, how an axonemal complex is able to modulate the localization of cytokinesis proteins at the new FAZ tip, and why this only occurs in one life cycle stage, is currently unclear.

In addition to recruiting TbPLK and TbAUK1 to the new FAZ tip, BioID studies in procyclic *T. brucei* show that TOEFAZ1/CIF1 interacts with, or is a near neighbor of, dozens of other proteins, including FAZ and microtubule–associated proteins (Zhou et al., 2016b, 2018a; Hilton et al., 2018). Many interactors are cytokinesis proteins, and TOEFAZ1/CIF1 is required for at least some of these to localize to the new FAZ tip (Figure 6C). For example, the kinetoplastid-specific protein phosphatase, KPP1, colocalizes with TbPLK throughout the cell cycle and with TOEFAZ1/CIF1 at the new FAZ tip (Zhou et al., 2016b; Hilton et al., 2018). KPP1 depletion substantially depleted TOEFAZ1/CIF1 and TbPLK and/or prevented TbPLK localization to the new FAZ tip (Hilton et al., 2018; Zhou et al., 2018b) and resulted in defects in duplicating and segregating flagellum-associated cytoskeletal structures (Zhou et al., 2018b). Post-mitotic cells with prematurely detached flagellar connectors accumulated, and cytokinesis was delayed or inaccurate. Increased phosphorylation of TbPLK substrate Centrin2 (de Graffenried et al., 2013) was also observed, suggesting that KPP1 opposes TbPLK action, either by dephosphorylating activatory T loop phosphorylation of TbPLK, or by dephosphorylating TbPLK substrates (Zhou et al., 2018a).

TOEFAZ1/CIF1 also localizes the kinesin, KLIF (Kinesin Localized to the Ingressing Furrow) to the FAZ tip and furrow (Hilton et al., 2018; Zhou et al., 2018a; Zhang et al., 2019a). KLIF depletion inhibits the latter stages of procyclic furrowing, but although it slows bloodstream form proliferation, it does not discernibly affect cytokinesis (Zhang et al., 2019a). The coiled coil protein, FRW1, also interacts with TOEFAZ1/CIF1, localizing to the FAZ tip, ingressing furrow, and the posterior cell tips and/or cytoplasmic bridge at abscission in procyclic trypanosomes, but exhibits a punctate localization in the middle of bloodstream form cells (Zhang et al., 2019a). Surprisingly, in the procyclic form, almost total FRW1 depletion did not affect proliferation, suggesting it may be non-essential for cytokinesis (Crozier et al., 2018; Zhou et al., 2018a), while in the bloodstream form, just moderate FRW1 depletion rapidly inhibited cytokinesis initiation (Zhang et al., 2019a).

TOEFAZ1/CIF1 also interacts with cytoskeleton remodeling proteins, including the katanin subunit, KAT80, and a coiled coil protein, PAVE1 (Posterior and Ventral Edge Protein 1). KAT80 and its associated KAT60 subunits are required for furrow ingression in procyclic and bloodstream trypanosomes, most likely due to a role in severing and remodeling microtubules (Casanova et al., 2009; Benz et al., 2012; Zhou et al., 2018a). PAVE1 is found at the posterior end of procyclic cells (and at both daughter cell ends in dividing cells), enriched on the ventral side, and also localizes to the cleavage fold and ingressing furrow (where it colocalizes with TOEFAZ1/CIF1). It is required for the tapering of daughter cell posterior ends (Hilton et al., 2018). Two translationally controlled tumor protein (TCTP) paralogues may also play a role in posterior end remodeling during procyclic form cytokinesis (Jojic et al., 2018a) but appear to be required for cytokinesis initiation in bloodstream form trypanosomes (Jojic et al., 2018b). The cytoskeleton-associated protein, AIR9, and the giant FAZ intermembrane staple protein, FAZ10, are also putative TOEFAZ1/CIF1 interactors (Hilton et al., 2018; Zhou et al., 2018a). AIR9 regulates posterior end microtubule extension and nucleus positioning in procyclic trypanosomes, and controls cleavage furrow placement accuracy in the bloodstream form (May et al., 2012), while FAZ10 plays roles in cell morphogenesis, flagellum attachment, kinetoplast and nucleus positioning, and optimal timing and placement of the cleavage furrow in both forms (Moreira et al., 2017).

TOEFAZ1/CIF1 therefore appears to be a critical scaffolding protein that dynamically interacts with many proteins required to signal and effect cytokinesis, thereby integrating the structural pre-requisites of cytokinesis (e.g., duplicating the flagellum and its associated structures/organelles) with the signaling molecules that coordinate cell division and the cytoskeleton remodeling proteins that effect it.

In addition to TOEFAZ1/CIF1 and its binding partners, most of which localize to structures (new FAZ tip, ingressing furrow, posterior cell tip) consistent with their cytokinesis function, there are various other essential cytokinesis proteins that are cytoplasmic. These seem to play specific and direct roles in cytokinesis, since their depletion and/or overexpression specifically affects cytokinesis without affecting earlier cell cycle events such as basal body segregation, which would have knock-on effects on cytokinesis. However, it is likely that these proteins operate upstream of, or perhaps in different pathways from, TOEFAZ1/CIF1. For example, the essential trypanosome receptor for activated C kinase (TRACK) scaffold protein, is critical for cytokinesis, with its depletion arresting procyclic cells mid-furrow (similar to KLIF depletion) and bloodstream parasites prior to furrow ingression (Rothberg et al., 2006). TRACK interacts (at least *in vitro*) with Rho-related protein, TbRHP, which is also required for progression through cytokinesis in both procyclic and bloodstream *T. brucei* (Abbasi et al., 2011). Both proteins mainly localize to the cytosol, where TRACK associates with EF1α in monosomes and polysomes; TRACK depletion reduces translation efficiency and renders trypanosomes hypersensitive to the translational inhibitor, anisomycin, suggesting that ongoing translation is required to complete cytokinesis (Regmi et al., 2008). Further, the cytosolic NDR kinases, PK50 and PK53, appear to regulate furrowing initiation and the latter stages of furrow ingression, respectively, in bloodstream form trypanosomes (Ma et al., 2010). While NDR kinases in other organisms are activated by a MOB (Mps One Binder) binding partner, and trypanosome MOB1 proteins are essential for the accuracy (procyclic) and completion (bloodstream form) of furrowing (Hammarton et al., 2005), PK50 and PK53 are MOB1-independent (Ma et al., 2010). Consistent with this, NDR kinases were reported as potential interactors of TOEFAZ1/CIF1, while the also cytosolic MOB1 proteins were not (Hilton et al., 2018). The cytosolic GTPase, ARL2, which regulates α-tubulin acetylation, and ESAG4/ESAG4-like adenylyl cyclases are also required for the latter stages of furrow ingression in the bloodstream form (Price et al., 2010; Salmon et al., 2012), and phosphodiesterases (PDEs) may promote abscission (de Koning et al., 2012).

#### T. cruzi

*T. cruzi* cells differ structurally from *T. brucei* and *Leishmania* since they possess an endo/exocytic cytostome-cytopharynx complex, associated with a triplet of microtubules running from beneath the cytostome membrane to the cell posterior and a microtubule quartet linking the cytostome with the flagellar pocket. The cytostome-cytopharynx complex and the microtubule triplet disassemble during G2 phase and reassemble during cytokinesis, guided by the cytostome microtubule quartet (Alcantara et al., 2017). However, while there are additional cytoskeleton components and the order of organelle replication differs in *T. cruzi* compared to *T. brucei* (Elias et al., 2007), the overall process of cytokinesis appears similar, with a furrow ingressing from the anterior to the posterior. Little is known about the molecular regulation of *T. cruzi* cytokinesis, although colchicine, which blocks microtubule polymerization, the protein phosphatase 1 (PP1)-inhibitor, calyculin, and the Casein Kinase 2 (CK2) inhibitor, emodin, inhibit epimastigote cytokinesis (Orr et al., 2000; Potenza and Tellez-Inon, 2015; De Lima et al., 2017). However, *T. cruzi* AUK1 is apparently not required for cytokinesis (Fassolari and Alonso, 2019).

#### *Leishmania* spp.

*Leishmania* cell division differs from that of *T. brucei* in several ways. G2 phase is much briefer and the order of nucleus and kinetoplast division varies according to the species; some species use more than one route to divide (Ambit et al., 2011; Minocha et al., 2011; Wheeler et al., 2011). Further, in promastigotes, the flagellum continues to grow from one cell cycle to the next and significant morphological changes occur during the cycle, with cells elongating during G1 phase and then shrinking and broadening during mitosis, prior to cytokinesis. A cleavage cleft forms along the longitudinal axis of the cell, with the furrow progressing from the anterior to the posterior with bilateral symmetry (Figure 8B). Abscission then occurs, but only in 90% cells in *L. mexicana*; the remaining 10% re-enter the cell cycle, replicating as doublets (Wheeler et al., 2011). Little is known about cytokinesis regulators in *Leishmania* promastigotes; only Aurora kinase has been linked to a possible role in cytokinesis, in addition to its roles in mitosis (Chhajer et al., 2016). However, actin dynamics are important, influencing flagellar pocket division, basal body/kinetoplast segregation and vesicular movement, with defects in these processes in turn inhibiting cytokinesis furrowing (Tammana et al., 2010). Further, several microtubule-severing agents localize to the cleavage furrow (LmjKAT106 and LmjKAT80) or midbody (LmjFID), suggesting that they sever microtubules during cytokinesis (Casanova et al., 2009). However, *Leishmania* do not have a spastin ortholog, highlighting differences in microtubule severing compared to *T. brucei*.

In contrast to promastigotes, *Leishmania* amastigotes are intracellular and morphologically rounded with only a vestigial flagellum. KHARON, a kinetoplastid-specific protein, involved in trafficking flagellar membrane proteins, is important for cytokinesis specifically in amastigotes grown in macrophages; *KHARON* null mutants are viable as promastigotes and axenic amastigotes, but exhibit flagellum and morphological aberrations and arrest in cytokinesis in macrophages (Tran et al., 2013, 2015; Santi et al., 2018). Similarly, knockout of a centrin gene in *L. donovani*, was only lethal in amastigotes but not promastigotes, demonstrating failure of basal body duplication and cytokinesis (Selvapandiyan et al., 2004).

## Cytokinesis in the Alveolata

### *Tetrahymena* spp. and *Balantidium coli* (Phylum Ciliophora): Hippo Pathway Signaling, Microtubule Remodeling, and Ciliary Beating Regulate Tandem Duplication

**Figure 9:**
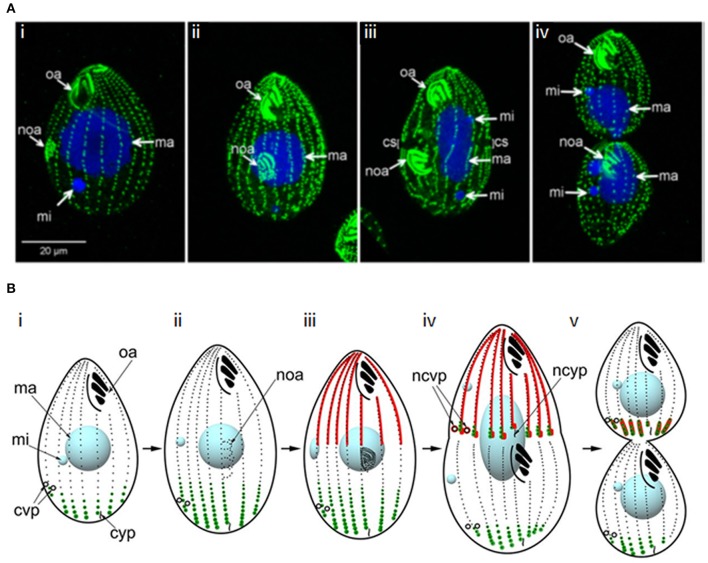
Cytokinesis in *Tetrahymena*. **(A)** Immunofluorescence images of a dividing *Tetrahymena* cell. Basal bodies are labeled with anti-centrin 205H antibody (green); DNA is stained with DAPI (blue); oa: oral apparatus; noa: new oral apparatus; mi: micronucleus; ma: macronucleus; cs: cortical subdivision. (i,ii) early stages of cell division highlighting development of the new oral apparatus; (iii) early stage of amitosis (elongated macronucleus)—the micronuclei have already completed mitosis and the cortical subdivision is visible; (iv) late cytokinesis–amitosis has completed. Adapted and republished with permission of Genetics Society of America from Jiang et al. (2017); permission conveyed through Copyright Clearance Center, Inc. **(B)** Schematic of the determination of cleavage site positioning by the localisations of two Hippo-kinases, Elo1 (green) and Cda1 (red), which sequentially inhibit segmentation at the posterior and anterior of the dividing cell, respectively. Mob1 shares an identical localization with Elo1, suggesting it might activate Elo1. However, it also colocalizes with Cda1 at the posterior end of the anterior daughter cell, with Mob1 depletion phenocopying Cda1 depletion, suggesting it may operate in both pathways. (i) cell in interphase; (ii, iii) early and late in the development of the new oral apparatus; (iv) early amitosis; (v) late cytokinesis. cvp, contractile vacuole pore; ncvp, new contractile vacuole pore; cyp, cytoproct; ncyp, new cytoproct. Adapted and republished with permission of Genetics Society of America from Jiang et al. (2019); permission conveyed through Copyright Clearance Center, Inc.

Ciliates are structurally complex, with micro-and macronuclei, an oral (feeding) apparatus, contractile vacuole pores, a cytoproct (a site of exocytosis and membrane recycling), and an intricate polar array of surface cilia. Cilia align in longitudinal rows alongside longitudinal microtubule bundles, and their basal bodies are associated with transverse and postciliary bands of microtubules. Most ciliates are free-living and non-pathogenic. However, certain *Tetrahymena* species infect fish, insects or dogs (Jerome et al., 1996; Lynn et al., 2000; Chettri et al., 2009) and *Ichthyophthirius multifiliis* infects freshwater fish (Coyne et al., 2011), while *Balantidium coli*, the largest known protozoan (25–200 μm), commonly found in the pig intestine, is the only known human-infective ciliate, causing balantidiasis (Schuster and Ramirez-Avila, 2008). Ciliates divide by transverse binary fission (Figure 9A). Following tandem duplication of the cortex, a furrow forms at the midpoint of the cell's anterior-posterior axis, constricting due to the presence of a divergent contractile ring. The most detailed studies of ciliate cell division have been performed in *Tetrahymena* spp., although other work indicates that *Nassula* (Tucker, 1971) and *Paramecium* (Jurand and Selman, 1969) also use a non-myosin II contractile ring to divide. Despite *B. coli* being a human pathogen, its cell division has been little studied, perhaps due to difficulties with its laboratory culture (Schuster and Ramirez-Avila, 2008; Nilles-Bije and Rivera, 2010).

*Tetrahymena* replication begins with basal body duplication and elongation of the longitudinal ciliary rows, following which a new oral apparatus is constructed in the posterior half of the cell. Mitosis of the micronucleus follows and a gap (the cortical subdivision) appears in the ciliary rows just anterior to the new oral apparatus, demarcating the newly-forming daughter cells. This region is free from cilia, basal bodies and their associated microtubules, but initially, longitudinal microtubule bundles remain. The macronucleus then divides amitotically, with the two resultant macronuclei being positioned either side of the cortical subdivision. Furrow ingression follows, with the concomitant severing of longitudinal microtubule bundles, resulting in the formation of new cortical ends; new contractile vacuole pores and cytoproct are constructed at the posterior end of the anterior daughter cell. To complete cytokinesis, a thin residual cytoplasmic bridge between the daughter cells must be cleaved; opposing rotational forces exerted by ciliary beating (“rotokinesis”) may contribute to this (Brown et al., 1999a).

The cortical subdivision is placed at the cell's midpoint by the joint actions of two opposing Hippo kinase signaling pathways (Figure 9B). The Hippo/MST2-like kinase, CDA1, and its putative binding partner and substrate, MOB1, exclude divisional activities in the anterior half of the cell, while a second Hippo kinase, ELO1, prevents division in the posterior half (Tavares et al., 2012; Jiang et al., 2017, 2019). Beneath the cortex at the division site, is a contractile ring containing actin, but no myosin II or septins (Yasuda et al., 1980; Hirono et al., 1987; Wloga et al., 2008). However, actin appears to play an indirect role in cytokinesis. It is found at multiple cellular sites in *Tetrahymena* and is required for phagocytosis and motility, leading to secondary effects on cytokinesis (Ohba et al., 1992; Brown et al., 1999a,b; Hosein et al., 2003; Williams et al., 2006). Indeed, inhibiting actin polymerization with Lantrunculin A does not prevent cytokinesis (Shimizu et al., 2013) and the actin-modulator, actin-depolymerization factor (ADF)/cofilin is not required for cytokinesis (Shiozaki et al., 2013). However, a number of actin-binding proteins localize to the division site (Edamatsu et al., 1992; Numata et al., 2000; Numata and Gonda, 2001; Shirayama and Numata, 2003), and some have been shown to be important for cytokinesis (Numata and Gonda, 2001; Wilkes and Otto, 2003). Further, repetitive protein p85 localizes to the furrow in a calmodulin/calcium-dependent manner; inhibition of this interaction inhibits contractile ring formation and furrowing (Gonda et al., 1999; Numata et al., 1999).

Tubulin dynamics and modifications also seem to be important for *Tetrahymena* cytokinesis. Polyglycylated β-tubulin is found in cilia, and at a lower level, in all cortical microtubules. Reduced polyglycylation results in ciliary paralysis (due to absence of the central microtubule pair and other microtubule defects) (Xia et al., 2000; Thazhath et al., 2002) and chains of incompletely separated daughter cells. While a lack of ciliary beat can inhibit cytokinesis completion (e.g., as in kinesin-II null mutants) (Brown et al., 1999b), other defects (disrupted cortical rows, and longitudinal microtubule bundles that continued to grow into the fission area or were not severed) were noted, suggesting an additional physical inhibition of furrowing (Thazhath et al., 2002). Katanin null mutants phenocopy β-tubulin mutations that prevent polyglycylation (Thazhath et al., 2002), suggesting katanin cleaves longitudinal microtubule bundles at the division site (Sharma et al., 2007).

### The Apicomplexa–Multiple Modes of Budding and/or Hijacking of Host Cell Mitotic Machinery

Apicomplexans comprise four major clades: the Coccidia, Plasmodia, Piroplasmida, and Cryptosporidia, and encompass a number of important human/animal pathogens. They infect a wide range of host cells, mostly residing within a parasitophorous vacuole. The apicomplexan cell (reviewed in Morrissette and Sibley, 2002a) is surrounded by a pellicle, comprised of the plasma membrane and the inner membrane complex (IMC), with apical and basal complexes at either end. The inner membrane complex consists of flattened alveoli associated with cytoskeletal elements including the actin-myosin motor complex (required for motility and invasion) and subpellicular microtubules (Dubremetz and Elsner, 1979; Adams and Todd, 1984; Kono et al., 2012; Dubey et al., 2017). The apical complex has roles in secretion and invasion, and its apical polar ring acts as an MTOC (Hu et al., 2002b), while the basal complex is a mobile and contractile proteinaceous ring important for mitosis and cytokinesis (Gubbels et al., 2006; Hu et al., 2006; Hu, 2008). Apicomplexans also contain a nucleus surrounded by endoplasmic reticulum, a mitochondrion, a Golgi, an apicoplast (a vestigial, non-photosynthetic plastid unique to this phylum), and two further MTOCs: the centrioles or centriolar plaque, and the basal bodies (Francia et al., 2015). In coccidians, two parallel centrioles form a centrosome, which is associated with the centrocone, a specialized invagination of the nuclear envelope that anchors the intranuclear mitotic spindle and plays key regulatory roles in budding (Sheffield and Melton, 1968). *Plasmodia* instead have a centriolar plaque within the nuclear envelope that nucleates the spindle microtubules (Francia and Striepen, 2014). Basal bodies are formed solely in male gametes, the only flagellated life cycle stage.

Apicomplexans display great flexibility in cell division (Francia and Striepen, 2014), exhibiting a vast range of proliferative scale, with a single parasite replicating to form just two or up to tens of thousands of daughters, depending on the species, life cycle stage and host. The apicomplexan cell cycle comprises just three main phases: G1, S, and M; G2 phase is very brief or absent (Radke et al., 2001). Some daughter cell components (e.g., centrosome, Golgi, nucleus, apicoplast, mitochondrion) arise through the duplication and division of mother cell organelles, while others (e.g., apical and basal complexes and inner membrane complex/cytoskeleton) are assembled *de novo* (Nishi et al., 2008). Apicomplexans divide by budding, assembling daughter cells either deep inside or at the plasma membrane of the mother cell. The appearance of the daughter inner membrane complex scaffolds in late S phase marks the start of budding (cytokinesis), and following completion of daughter cell assembly, daughters are cleaved from each other at the basal end via constriction of the basal complex (abscission) (Hu, 2008), and egress from the mother cell, acquiring her plasma membrane on the way (Hu et al., 2002a). The remaining mother cell components either disassemble during cytokinesis or are assimilated into a residual body after abscission (Hu et al., 2002a).

**Figure 10:**
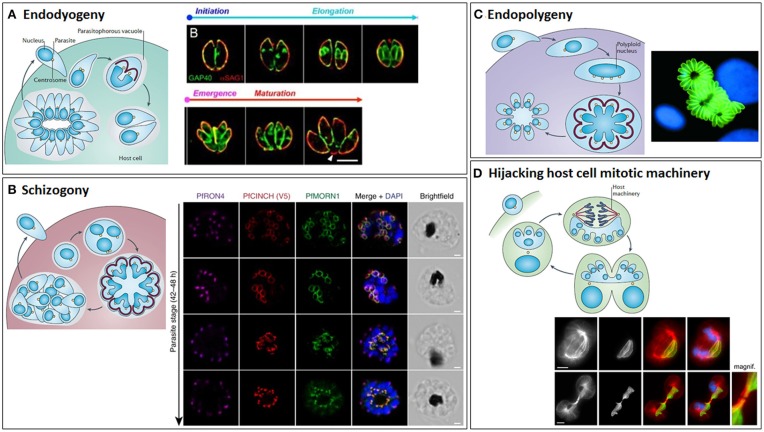
The different modes of cytokinesis in Apicomplexans. Apicomplexans may divide by endodyogeny **(A)**, schizogony **(B)**, endopolygeny **(C)**, or hijacking the host mitotic machinery **(D)**. Schematics of each mode of division [reprinted by permission from Springer Nature^©^ (Francia and Striepen, 2014)] are shown alongside corresponding fluorescent microscope images. **(A)** Endodyogeny, employed by *T. gondii* tachyzoites, is the simplest form of cell division, where just two daughter cells are constructed inside each mother cell within the parasitophorous vacuole. Daughter cells egress from the mother cell, but remain within the parasitophorous vacuole and undergo geometric expansion via further synchronous rounds of endodyogeny, generating rosettes of parasites connected by tubules radiating from the residual body. Subsequently, the replicated parasites lyse the parasitophorous vacuole membrane and egress from the host cell. Images show *Toxoplasma gondii* tachyzoites undergoing endodyogeny. Green: integral membrane protein, GAP40; red: plasma membrane protein, SAG1. The initiation and elongation of the daughter cell membrane can be seen, followed by emergence of the daughter parasites from their mothers and their subsequent maturation. Scale bar: 5 μm. Adapted from (Ouologuem and Roos, 2014). **(B)** Schizogony (employed by *Plasmodium* spp. in their mammalian hosts, *Eimeria* spp. in host intestinal cells and *Babesia* spp. in nucleated blood cells) involves repeated rounds of DNA replication and mitosis in the absence of budding, followed by a final round of division culminating in a synchronous budding event at the surface of the mother cell. Again, this is followed by lysis of the parasitophorous vacuole and egress from the host cell. Images taken from timecourse of segmentation in *Plasmodium falciparum*. Airyscan superresolution microscopy of a segmenting schizont showing inner membrane complex (PfMORN1, green) and basal complex assembly (PrCINCH, red) alongside staining for the rhoptry neck protein, PfRON4 (magenta), and DNA (DAPI, blue). Note the widening of the basal complex ring as it extends past the nucleus and then its tapering as it reaches the basal end of the parasite. Scale bars: 1 μm. Taken from Rudlaff et al. (2019). Note that *T. gondii* utilizes endopolygeny to divide within the cat host, which is similar to schizogony, involving repeated rounds of S/M phases, but followed by a final round of cell division and synchronous budding deep within the mother cell. **(C)** *Sarcocystis* spp. divide by a variant of endopolygeny, involving multiple rounds of S phase in the absence of mitosis, generating a giant polyploid nucleus, prior to a final synchronous round of cell division with mitosis and budding. In some cases, the merozoites produced remain within the host cells and undergo additional rounds of division, this time asynchronously. Image shows schizonts of *Sarcocystis neurona* (green) (Daniel K. Howe). **(D)** *Theileria* spp. (and some *Eimeria* spp.) utilize the host cell's mitotic machinery to divide, with parasite schizonts associating with host centrosomes and spindle microtubules such that they span the metaphase plate and become trapped within the midbody, ensuring cleavage of the schizont and segregation to each host daughter cell at abscission. *Theileria* schizonts may also undergo merogony (as *Babesia* spp. and *Cryptosporidium* also do in red blood cells and epithelial cells, respectively) where they are packaged into individual merozoites that are released into the bloodstream and invade erythrocytes. Images show the segregation of a *Theileria annulata* schizont, highlighting its association with the host mitotic spindle microtubules (top) and cytoplasmic bridge/midbody (bottom). Left to right: anti-α-tubulin, anti-*Theileria annulata* surface protein 1 (TaSP1), anti-α-tubulin (red)/anti-TaSP1 (green) merge, anti-α-tubulin/anti-TaSP1/DAPI (blue) merge. Scale bars: 5 μm. Taken from von Schubert et al. (2010).

Depending on the species and their host, apicomplexans may suspend cytokinesis (undergoing just a nuclear cycle) and sometimes also mitosis, to achieve rapid successive rounds of DNA replication, before a final (usually synchronous) budding cycle assembles multiple daughters (Figure 10). For example, *T. gondii* tachyzoites usually replicate by endodyogeny, assembling just two daughter cells inside each mother cell (Figure 10A) (Sheffield and Melton, 1968; Hu et al., 2004), but employ endopolygeny within their definitive host, the cat (Ferguson et al., 2008). Endopolygeny involves multiple cycles of S/M phases in the absence of budding, forming a multinucleate cell, before a single synchronized round of mitosis and budding occurs deep within the mother cell. This is very similar to schizogony that is employed by *Eimeria* and *Plasmodium* spp. (Figure 10B); again, repeated rounds of S/M phases occur without budding, followed by a final round of division that ends with synchronous budding, but at the mother cell surface (Ferguson et al., 2008). A variation of endopolygeny occurs in *Sarcocystis* spp., whereby the nucleus undergoes multiple (five in *S. neurona*) rounds of S phase without mitosis, generating a large polyploid (32N) nucleus (Figure 10C). Multiple intranuclear mitotic spindles are assembled during S phase, which although always present, shrink back to mini spindles during interphase, allowing chromosome segregation/organization during each round of S phase, but without nuclear division. A final round of division proceeds (64N), culminating in a mass, synchronized karyokinesis and budding event yielding 64 haploid daughter cells (Vaishnava et al., 2005). In contrast to most apicomplexans, which exit their host cell following division, *Theileria* remains intracellular, exploiting host mitotic machinery to segregate between host cells during host cell division (Figure 10D) (von Schubert et al., 2010).

#### The Coccidia—*Toxoplasma gondii, Sarcocystis neurona*, and *Eimeria* spp.

The majority of apicomplexan cell division studies have been performed in *T. gondii* tachyzoites, which cause Toxoplasmosis, since they are experimentally tractable and divide by endodyogeny, the simplest form of apicomplexan division. Multiple rounds of daughter cell assembly occur synchronously within a single parasitophorus vacuole, forming rosettes, before the daughters lytically egress from the host cell. During S phase, a TgMORN1 (membrane occupation and recognition nexus repeat protein 1) ring forms around duplicated centrioles to initiate basal complex assembly followed by assembly of the conoids and apical rings, the inner membrane complex and subpellicular microtubules (Hu et al., 2002a, 2006; Gubbels et al., 2006; Ferguson et al., 2008; Hu, 2008; Nishi et al., 2008). Centrosome splitting is crucial for daughter cell construction; if centrosomes are replicated but not segregated, only a single daughter scaffold is assembled (Chen and Gubbels, 2013). A fiber of striated fiber assemblin (SFA) proteins then extends from each centrosome, which is essential to position the daughter apical complexes (Francia et al., 2012). As the inner membrane complex extends during the rest of the cell cycle, the basal complex migrates distally, enveloping the replicated and segregated organelles, widening to migrate over the daughter cell nucleus post-mitosis, and then undergoing centrin-mediated contraction to form the tapered basal end (Hu et al., 2002a; Hu, 2008; Nishi et al., 2008). However, all daughter cells in a rosette remain connected within the parasitophorus vacuole by tubules radiating from the residual body that not only contribute to the spatial organization of the rosette (Muniz-Hernandez et al., 2011), but ensure that daughters share cytoplasm until they egress from the host cell (Frenal et al., 2017). Further constriction of the basal complex occurs post-emergence from the mother cell (Hu, 2008), and this, or alternatively, degradation or recycling of mother cell material (Ouologuem and Roos, 2014) and/or mechanical stress from daughter cell motility as they egress from the mother (Lorestani et al., 2010) has been proposed to lead to completion of abscission. Following emergence, the daughter cell inner membrane complexes continue to mature; proteolytic processing of the TgIMC1 protein confers increased stability to the inner membrane complexes (Mann et al., 2002) and the inner membrane complexes expand with the incorporation of maternal inner membrane complex proteins recycled from the residual body (Ouologuem and Roos, 2014).

The *Toxoplasma* cytoskeleton is thus critical for cell division. Active microtubule polymerization is essential for mitosis and inner membrane complex elongation during budding (Shaw et al., 2000; Morrissette and Sibley, 2002b). Further, many other cytoskeletal and cytoskeleton-associated proteins display dynamic and hierarchical localizations during inner membrane complex deposition in daughter cells (Beck et al., 2010; Anderson-White et al., 2011; Chen et al., 2015, 2017), although only ISP2 has been shown to be required for cell division (Beck et al., 2010). Actin, Formin-2 (TgFRM2) and various non-myosin II myosins are also important for *Toxoplasma* cell division, with roles in centrosome positioning, apicoplast segregation, turnover of mother cell organelles during daughter cell budding, and residual body formation (Shaw et al., 2000; Andenmatten et al., 2013; Jacot et al., 2013; Frenal et al., 2017; Stortz et al., 2019). Additionally, actin has been proposed to work in concert with the class XXIII (VI-like) myosin, TgMyoJ, and TgCentrin2 to bring about basal complex constriction, although a direct interaction between these proteins has not yet been demonstrated (Frenal et al., 2017). Another myosin, the class XIV TgMyoC, also localizes to the basal complex, and overexpression of its splice variant, TgMyoB, causes defects in cytokinesis (Delbac et al., 2001). Finally, TgMORN1 localizes to the basal complex (as well as the apical ring and the centrocone) and is important for cytokinesis (Gubbels et al., 2006; Hu et al., 2006), with its role likely conserved across the Apicomplexa (Ferguson et al., 2008). Overexpression of TgMORN1 results in *Toxoplasma* parasites being unable to duplicate their centrocone, segregate their nucleus or to bud (Gubbels et al., 2006), while *TgMORN1* knockout affects basal complex organization, Golgi replication, apicoplast segregation, and cytokinesis (Heaslip et al., 2010; Lorestani et al., 2010). A haloacid dehalogenase (HAD) phosphatase, TgHAD2a, which interacts with TgMORN1 at the basal complex, is also required for correct basal complex organization and its constriction at cytokinesis (Engelberg et al., 2016).

Various *Toxoplasma* cell cycle regulators have been described. Apicomplexans use a “just in time” approach to gene transcription, with two main waves of transcription: growth and house-keeping genes are transcribed in G1 phase, while daughter cell assembly and cell division genes are transcribed in S phase (Behnke et al., 2010; Gaji et al., 2011). Ten Crks have been identified, along with seven cyclins (Cycs) from the L, P, H, and Y classes, but these are rather atypical, with expression of some Crks (TgCrk4, TgCrk5, and TgCrk6), but only one cyclin (TgCycY) oscillating through the cell cycle (Alvarez and Suvorova, 2017). Only one Crk, TgCrk1, has been shown to date to regulate cytokinesis, although depletion of TgCrk4 or TgCrk6, which regulate centrosome duplication and mitosis, respectively, also has downstream effects on budding (Alvarez and Suvorova, 2017). TgCrk1 is a Cdk11 family member, whose orthologs regulate mRNA synthesis and maturation; it is localized in the nucleus and, along with its partner, CycL, is required for daughter bud formation, suggesting perhaps that this CDK complex regulates the S phase wave of transcription/splicing. Depletion of TgCrk11 or TgCycL results in improperly formed daughter cell TgMORN1 rings, leading to defective basal complexes, disorganized inner membrane complex scaffolds and deformed apical ends (Alvarez and Suvorova, 2017).

Two Aurora family kinases, TgArk1 and TgArk3, also play important roles in *Toxoplasma* cell division (Suvorova et al., 2015; Berry et al., 2016, 2018). Dominant negative mutants of chromosomal passenger complex kinase TgArk1 result in an early mitotic block and a knock-on effect on cytokinesis. A “Russian doll” phenotype ensues, whereby multiple rounds of centrosome duplication and budding occur, but budding does not complete due to the block in mitosis, and multiple layers of incomplete inner membrane complexes form inside each other (Berry et al., 2018). TgArk3 [confusingly, originally named TgArk1 (Suvorova et al., 2015)] is first expressed in S phase and localizes to the outer centrosome core, and later, during mitosis/cytokinesis, also localizes to one side of the forming daughter cytoskeletons (Suvorova et al., 2015; Berry et al., 2016). TgArk3-depleted parasites undergo karyokinesis, but show an impairment in daughter cell budding, resulting in reduced replication rates, correlating with the known role of the centrosome outer core in controlling budding (see below). TgArk3-depletion also results in shorter daughter parasites, defective rosette formation and impaired invasion and virulence (Berry et al., 2016). Further cell cycle regulators undoubtedly remain to be identified. A forward genetics chemical mutagenesis screen isolated fifty temperature sensitive growth mutants with defects in budding, accompanied or not by defects in karyokinesis (Gubbels et al., 2008). However, only some of the genes affected in the mutants are known and few [e.g., Nek1 (mutant V-A15), see below] have been studied in detail.

The *Toxoplasma* centrosome is key to cell division, directing mitotic spindle formation via the centrocone and regulating daughter cell bud assembly, and plays a critical regulatory role in determining which division cycle occurs (Suvorova et al., 2015). It is comprised of two proteinaceous cores. The outer core, distal from the nucleus, contains TgCentrin1, the centrin-binding protein, TgSfi1, cartwheel protein TgSas-6, γ-tubulin, TgArk3 and during cytokinesis, also TgCep250. Depletion of TbSfi1 leads to reduced duplication and/or loss of outer cores, accompanied by uncontrolled replication of the inner core and centrocone and inhibition of budding (Suvorova et al., 2015). The inner core, located close to the centrocone, is comprised of orthologs of the CEP250/C-Nap centrosomal protein family, including a proteolytically processed form of TgCep250, present throughout the cell cycle. Depletion of TgCep250 disrupts the connection between inner and outer cores of the daughter centrosome, resulting in inner core loss and disruption of mitosis, without affecting outer core duplication or budding (Suvorova et al., 2015; Chen and Gubbels, 2019). Further, a large coiled-coil protein, TgCep530, localizes to the interface of the inner and outer cores and appears to coordinate nuclear packaging into daughter cells (Courjol and Gissot, 2018). Thus, the centrosome inner and outer cores appear to control mitosis and budding, respectively, with several protein kinases in addition to TgArk3 (discussed above) involved in this regulation. TgNEK1 localizes to the proximal ends of duplicating centrosomes and regulates centrosome splitting and therefore daughter cell scaffold assembly (Chen and Gubbels, 2013), and the calcium-dependent protein kinase, TgCDPK7, regulates centrosome duplication and integrity, and daughter cell orientation, amongst other roles (Morlon-Guyot et al., 2014). Further, the MAP kinase-like kinase, TgMAPK-L1, a candidate pericentriolar matrix protein that surrounds the TgCentrin1 outer core, is proposed to promote entry into the budding cycle and to suppress the nuclear cycle (Suvorova et al., 2015). Its depletion results in over-amplified centrocones and inhibition of basal complex ring formation, resulting in multiple rounds of S/M phases, without cytokinesis or accompanied by defective budding. In the absence of TgMAPK-L1 activity, or if the outer core is faulty or not assembled (e.g., as seems likely in the early division cycles of endopolygeny or schizogony, although this has not yet been demonstrated), the nuclear cycle is the default (Chen and Gubbels, 2015; Suvorova et al., 2015). Further, many integral centrosomal proteins have been identified, which are likely to be important for centrosome function and accurate budding (Morlon-Guyot et al., 2017). Ubiquitination may also play a role in controlling inner/outer core stoichiometry and coupling of mitosis and cytokinesis (Dhara et al., 2017). In addition, a number of other proteins have functions linked to controlling the switch between endodyogeny and endopolygeny. The inner membrane complex components, IMC14, IMC15, and ISP2, as well as the trafficking GTPase, Rab6, and the lipid storage/membrane biosynthesis stimulatory protein, TgNCR1, have been shown to limit the number of *T. gondii* offspring to two per mother cell (Stedman et al., 2003; Beck et al., 2010; Lige et al., 2011; Dubey et al., 2017); IMC14 depletion also affects synchrony of cell division (Dubey et al., 2017).

Far less is known about cell division in other coccidians, although imaging studies have revealed key differences compared to *T. gondii*. *Sarcocystis neurona*, the causative agent of equine protozoal myeloencephalitis in horses, replicates via a variant of endopolygeny, as discussed above, which can occur asynchronously within a single host cell i.e., the resultant merozoites sometimes remain within their original host cell and undergo another round of division (Speer and Dubey, 2001). Little is known about the molecular regulation of *Sarcocystis* cell division. Analysis of the *S. neurona* kinome has shown that most kinases discussed above are conserved (Murungi and Kariithi, 2017), but none have been functionally characterized. Further, while budding superficially resembles that in *Toxoplasma*, some inner membrane complex proteins may have differential functions compared to their *T. gondii* homologs (Dubey et al., 2017). *Eimeria* spp., which cause coccidiosis in poultry and ruminants, divide via schizogony within intestinal cells of their host. The first generation of schizogony involves sporozoites transforming to schizonts, while subsequent generations involve merozoites undergoing schizogony. Species-specific differences in schizont size between the first and second generations have been noted (Dubremetz and Elsner, 1979; Gregory et al., 1989; Ferguson et al., 2007). Further, *E. vermiformis* first generation merozoites bud randomly from the schizont, while second generation merozoites align in parallel before unidirectional budding (Adams and Todd, 1984). In *E. crandallis* and *E. bakuensis*, following second generation schizogony, dividing parasites appear to stretch across the host cell mitotic spindle (Gregory et al., 1987, 1989), similar to *Theileria* (see below). However, as with *Sarcocystis*, molecular detail is lacking; the CDK inhibitor, flavopiridol, inhibits schizont development (Engels et al., 2010), but functional analyses of CDKs/cyclins have not yet been performed (Kinnaird et al., 2004; Fernandez et al., 2012).

#### The Plasmodia

In *Plasmodium* spp., the causative agents of malaria, there are added complexities to cell division, with staggering differences of scale associated with their intricate life cycles (Gerald et al., 2011), where they are spread between animal hosts by mosquito vectors. There are four distinct cytokinesis events within the malaria life cycle. Within the mosquito, microgametocytes undergo three sequential rounds of S/M phase (forming eight basal bodies and flagellar axonemes *de novo*) in rapid succession (Billker et al., 1998). Chromatin then condenses, axonemes become motile, vesicles containing membranolytic proteins are secreted and a single mass and very rapid (8–12 min) cytokinesis event, known as exflagellation, occurs, whereby eight microgametes are formed as axonemes swim out of the residual gametocyte body, each dragging a condensed haploid genome attached to the basal body with it. Subsequently within the mosquito, sporogony (10–11 rounds of S/M phases over ~10 days) occurs, generating syncytial cells containing thousands of nuclei before a mass synchronous budding event occurs, releasing tens of thousands of infectious haploid sporozoites. Two more mass budding events occur within the mammalian host, at the end of schizogony in hepatocytes and erythrocytes, generating merozoites. Within hepatocytes, syncytia containing tens of thousands of nuclei are formed. The parasite plasma membrane invaginates (cytomere stage), forming spheres within the parasitophorous vacuole, each containing multiple nuclei, with branches of the apicoplast at the periphery and clumps of mitochondrion in the center. Regular constrictions then appear along the apicoplast's length, and mitochondrial protrusions reach out to each nucleus. The apicoplast then divides synchronously, followed by the mitochondrion, and then cytokinesis completes, with further invagination of the plasma membrane to generate individual haploid merozoites, which are released into the bloodstream in parasite-filled vesicles known as merosomes (Sturm et al., 2006). Merosomes then disintegrate, releasing merozoites into the circulation, which then invade red blood cells. Blood stage schizogony (lasting ~48 h), in comparison, comprises just 3–4 rounds of asynchronous S/M phases before a synchronous budding event occurs to yield 16–32 daughter merozoites.

Several proteins are known to be essential for exflagellation in male gametocytes. Calcium dependent protein kinase 4 (CDPK4) activity is required at multiple points during microgamete development; a large myristoylated isoform is required for initiating DNA replication and mitotic spindle assembly while a small non-myristoylated isoform also supports spindle assembly and is essential for axonemal motility just prior to exflagellation (Billker et al., 2004; Fang et al., 2017). The CDPK4 substrate, SOC3, an axoneme-associated protein, appears to be the effector for driving axonemal motility and cytokinesis (Fang et al., 2017). A gametocyte and mosquito-stage-specific actin, PfACT2, is also required for axoneme motility and exflagellation (Deligianni et al., 2011). Further, the MAP kinase, PfMAP-2, an *in vitro* substrate of CDPK4 and PfNek1, and APC/C components CDC20 and APC3, are required for axonemal motility, although perhaps indirectly as depletion of these proteins also blocks chromatin condensation/karyokinesis (Dorin et al., 2001; Rangarajan et al., 2005; Tewari et al., 2005; Wall et al., 2018). Depletion of a serine/argine-rich (SR) protein kinase, SRPK (predicted to act as a pre-mRNA splicing factor), or of the protein phosphatase, PPM1, also completely blocks exflagellation, although the exact stage of microgamete development affected has not been determined (Tewari et al., 2010; Guttery et al., 2014; Robbins et al., 2017).

Little is known about the regulation of cytokinesis at the end of sporogony, with just the phosphatase, PTPLA, the cyclin, PfCYC3, and LAP (LCCL lectin domain adhesive-like proteins) family proteins having been implicated (Roques et al., 2015; Saeed et al., 2018, 2019). Further, nothing is known of the regulation of schizogony in hepatocytes, so all knowledge of cytokinesis at the end of schizogony comes from studies of the blood stages. A key regulator of schizont cytokinesis is the divergent cyclin PfCYC1, a cyclin that is most similar at the sequence level to cyclin H, yet when expressed in yeast, functions instead as a G1 cyclin (Robbins et al., 2017). PfCYC1 is expressed throughout the blood stage cell cycle, but its knockdown specifically inhibits schizont segmentation, with aberrant formation of daughter cell inner membrane complexes (Robbins et al., 2017). Although PfCYC1 can activate PfPK5 (the CDK1 homolog) *in vitro* (Le Roch et al., 2000), it does not seem to interact with PfPK5 *in vivo*, instead forming a complex with the CDK7 homolog, PfMRK, and its accessory protein, PfMAT1, reminiscent of the yeast and metazoan CDK7/Cyclin H/MAT1 complex that comprises transcription factor IIH. TFIIH promotes efficient RNA polymerase II transcription, but CDK7 also acts as a CDK-activating kinase in some organisms, linking transcription and cell cycle control. PfCYC1/MRK/MAT1 phosphorylates the RNA polymerase II C-terminal domain *in vitro*, and the activity of CYC1/MRK is increased by the presence of MAT1 (Chen et al., 2006; Jirage et al., 2010), but how exactly it regulates cytokinesis is unknown. The kinases PfPK7 and PfCRK5 are required for efficient merozoite generation from schizonts and optimal proliferation rates (Dorin-Semblat et al., 2008, 2013), but their exact role has also not been determined. However, they both localize to the nuclear periphery in schizonts and interact *in vitro*, suggesting they might operate in the same pathway (Dorin-Semblat et al., 2013).

Several structural proteins are also required for cytokinesis of blood stage parasites. Knockdown of the merozoite organizing protein, PfMOP, results in defective inner membrane complex formation and incomplete segmentation of merozoites. Since PfMOP localizes to the apical end of daughter cells and first appears early in schizogony, it may organize inner membrane complex formation (Absalon et al., 2016). Later, the actin, PfACT1, and its nucleator, Formin-2 (PfFRM2), are required for apicoplast segregation at the end of schizogony, with their disruption resulting in conglomerates of merozoites (Das et al., 2017; Stortz et al., 2019). Actin filaments connect apicoplasts during schizogony and are thought to allow their segregation and promote efficient cell separation at the end of cytokinesis. The coordinator of nascent cell detachment, PfCINCH, a basal complex protein, is also essential for correct segmentation of daughter cells and for daughter cells pinching off from the residual body (Rudlaff et al., 2019). PfCINCH colocalizes with PfMORN1 at the basal complex, and interacts with the *Plasmodium*-specific proteins, basal complex protein 1 (PfBCP1) and basal complex transmembrane protein 2 (PfBTP2); PfBTP1 also colocalizes with PfMORN1 and may be involved in the interactions between the invaginating parasite plasma membrane and the inner membrane complex during the latter stages of cytokinesis (Kono et al., 2016). Finally, a trafficking protein, PfSortilin, is essential for inner membrane complex and basal complex contractile ring formation and the generation of merozoites, also playing a role in apicoplast segregation (Hallee et al., 2018), although its *T. gondii* ortholog is not required for pellicle formation (Sloves et al., 2012).

#### The Piroplasmida—*Theileria* and *Babesia* spp.

Piroplasms are intracellular parasites, exclusively transmitted by hard ticks worldwide. Sporozoites from tick saliva are introduced into the vertebrate bloodstream, where they undergo different forms of asexual replication in various blood cells, depending on the species (Jalovecka et al., 2018). *Theileria* spp. infect cattle, invading and transforming leukocytes, causing pronounced pathology and high mortality rates. *Babesia* spp. cause Babesiosis, an emerging zoonosis and the most common blood disease of free-living animals, leading to abortions, reduced meat and milk production and even death. *Cytauxzoon* causes often fatal disease in cats, but has been little studied.

*Theileria* sporozoites are immotile, with a poorly developed apical complex. They invade monocytes and lymphocytes, shedding their coat and escaping from the host cell membrane surrounding them to replicate free in the cytoplasm by schizogony. Schizonts from many, but not all, *Theileria* spp. interfere with their host's mitogenic pathways, causing continuous replication and inhibition of apoptosis. Within transformed leukocytes, *Theileria* and the host replicate their DNA asynchronously, with parasite S phase occurring predominantly during early host cell mitosis (Irvin et al., 1982), but divide in synchrony. To ensure transmission to daughter host cells, schizonts integrate themselves into the host's mitotic machinery, associating with newly formed spindle microtubules (Hulliger et al., 1964; Seitzer et al., 2010; von Schubert et al., 2010), and spanning across the equatorial region of the cell at metaphase. At anaphase, following inactivation of host cell CDK1, the schizont scavenges active host cell polo-like kinase (Plk1) to its surface, allowing the schizont to associate closely with the central spindle (von Schubert et al., 2010). The middle of the schizont is trapped within the midbody during telophase, ensuring schizont cleavage and approximately equal segregation as host cells undergo abscission (von Schubert et al., 2010), which is important as continued parasite presence is required for host cell transformation (Hudson et al., 1985).

Various parasite proteins facilitate host cell microtubule interaction. The *T. annulata* surface protein, TaSP, interacts with host cell alpha and gamma tubulin, enabling parasite association with host cell centrosomes, mitotic spindle and midbody (Seitzer et al., 2010). A secreted protein, TaSE, also interacts with alpha tubulin, colocalising with the host cell centromere, mitotic spindle and midbody (Schneider et al., 2007). Further, the schizont membrane protein, p104, interacts with host microtubule plus-end-tracking protein (+TIP) End-binding protein 1 (EB1), likely directing growing microtubules to the schizont surface (Woods et al., 2013), and also with the microtubule-stabilizing + TIP protein CLASP1 (Huber et al., 2017). Finally, a novel schizont surface protein, TA03615, immunoprecipitates with EB1 and CLASP1, suggesting that it too might be involved in regulating schizont:microtubule interactions (Huber et al., 2017).

*Theileria* schizonts also undergo merogony (Shaw and Tilney, 1992), where they are packaged into individual merozoites. Following organelle replication, the forming merozoites bud, apical end first, in two or more synchronous waves, from the schizont surface. The schizont plasma membrane invaginates and a sublamellar basket of tubules extends from the apical to the basal end of each parasite, with a basal ring thought to constrict to separate the merozoite from the schizont. The leukocyte then ruptures, releasing merozoites into the bloodstream where they invade erythrocytes, transform into the piroplasm stage and undergo a limited number of asynchronous and little studied replication cycles, before some transform to gametocytes (Jalovecka et al., 2018). Upon uptake by a tick, gametocytes differentiate to gametes and then motile kinetes, which migrate to the salivary glands (Mehlhorn and Schein, 1976; Schein et al., 1977; Jalovecka et al., 2018) and rapidly replicate to form multinucleated syncitia within structures termed acini, before a poorly understood mass cytokinesis event occurs, giving rise to ~30–50,000 sporozoites per acinus (Fawcett et al., 1982).

In contrast, *Babesia* spp. multiply exclusively by merogony in red blood cells. Sporozoites injected by a tick bite are internalized into erythrocytes where they develop into ring stages or trophozoites and replicate freely and asynchronously in the cytoplasm, producing merozoites, which are released upon host cell rupture to invade other red blood cells. Little more is known about *Babesia* merogony or of the molecules that control it, although inhibitor studies suggest that CDKs are required (Nakamura et al., 2007). Indeed, despite piroplasms having the smallest kinomes of the Apicomplexans, with just 35 protein kinases in *B. bovis* and 37 and 38 in *T. annulata* and *T. parva*, respectively (Miranda-Saavedra et al., 2012), none have been functionally characterized. Within the tick, development of *Babesia* is mostly similar to that of *Theileria*. However, during sporogony, sporoblast maturation involves cytomere formation in some *Babesia* spp., which has not been observed in *Theileria* spp. (Jalovecka et al., 2018), and sporogony is asynchronous in *Babesia*, resulting in the continued release of sporozoites into tick saliva over several days (Yano et al., 2005).

#### The Cryptosporidia

*Cryptosporidium* spp., once thought to be coccidian parasites, are gregarines (Clode et al., 2015) which infect gut epithelial cells, causing diarrhea in humans and ruminants. They structurally differ from other apicomplexans, lacking an apicoplast and mitochondrial DNA (Bouzid et al., 2013). Sporozoites released from ingested sporulated oocysts invade host epithelial cells, becoming encapsulated in a parasite-modified host membrane on top of the epithelial cells, transforming into a trophozoite and replicating by merogony to release eight merozoites (O'Hara and Chen, 2011). However, in axenic culture, differentiation to trophozoites and merogony may occur within sporulated oocysts, with merozoites budding asynchronously from the oocyst membrane, with electron-dense collars at the bud site (Aldeyarbi and Karanis, 2016). Analysis of the *Cryptosporidium* kinome indicates that it lacks all aurora kinases and several CRKs, and it only possesses one NEK kinase (NEK1), suggesting divergent control. Recent advances in culturing (Wilke et al., 2019) should facilitate further studies of *Cryptosporidum* cytokinesis.

## Summary

Parasitic protozoans display an impressive array of cytokinesis mechanisms. These range from inaccurate and primitive cytofission in *Entamoeba* spp. to very precise and tightly regulated microtubule rearrangements that lead to furrow ingression in kinetoplastids, intricate assembly and segmentation of new daughter cells during apicomplexan budding, and ingenious hijacking of host mitotic machinery employed by *Theileria* and some *Eimeria* spp. However, to date, no protozoan parasite is known to use a contractile actomyosin ring, with most lacking myosin II. Even in *Entamoeba*, which does possess myosin II, there is no evidence for contractile actomyosin ring formation and only limited data to suggest that myosin II is important for cytokinesis in certain cell types (Majumder and Lohia, 2008; Krishnan and Ghosh, 2018). It remains to be seen whether myosin II-containing *Naegleria* spp. employ an actomyosin ring to divide, but other aspects of the cell cycle are divergent (Fritz-Laylin et al., 2010, 2011). Ciliates and some apicomplexans, which lack myosin II, however, do employ other types of contractile rings (Tucker, 1971; Yasuda et al., 1980; Hu, 2008). In *T. gondii* the basal complex ring contains non-myosin II myosins, which aid basal complex constriction (Delbac et al., 2001; Engelberg et al., 2016; Frenal et al., 2017), but *Plasmodium* spp. lack orthologs of some of these myosins, and the cytokinesis role of others is not conserved (Wall et al., 2019). Hence, myosins may not be involved in cytokinesis across the apicomplexa. Further, *Giardia* and *Trichomonas* spp. do not possess any functional myosins, relying on cytoskeletal elements/proteins and flagellar motility to bring about cytokinesis (Ribeiro et al., 2000; Benchimol, 2004b; Hardin et al., 2017). Flagellar/ciliary motility is also important for cytokinesis in *T. brucei* (Branche et al., 2006; Ralston et al., 2006; Zhang et al., 2019b) and *Tetrahymena* (Brown et al., 1999a), and during exflagellation in *Plasmodium* spp. (Billker et al., 1998).

It is not surprising, therefore, that molecular control of cytokinesis across the parasitic protozoans is also divergent. Canonical molecular cell cycle checkpoints are often absent; some key cell cycle regulators are present, but their functions are not always conserved and they may act in conjunction with parasite-specific regulators. The level of molecular control is also variable, from minimal in *Entamoeba* (Mukherjee et al., 2009; Grewal and Lohia, 2015) to highly intricate with built-in flexibility in the kinetoplastids and apicomplexans. However, despite the physical and molecular differences compared to model organisms, some aspects of cytokinesis are conserved. Vesicle delivery to the furrow, the plasma membrane lipid composition and changes in cortical tension are key for cytokinesis in *Giardia* and/or *T. brucei* (Lillico et al., 2003; Sheader et al., 2005; Rodgers et al., 2007; Fridberg et al., 2008; Stefanic et al., 2010; Paredez et al., 2011; Hardin et al., 2017; Schoijet et al., 2018). Further, protozoan parasites must cleave an intercellular cytoplasmic bridge containing cytoskeletal components that still links the daughter cells late in cytokinesis, to complete abscission, although midbodies have not been described in protozoan parasites, and in many protozoans, similar to metazoans, the rate of abscission is different from that of furrowing.

### Exploiting Cytokinesis for Drug Discovery

Given the essentiality and uniqueness of cytokinesis in protozoan parasites, key cytokinesis proteins have strong potential as novel drug targets. In particular, protein kinases are of interest, since kinases are druggable molecules (Urbaniak et al., 2012), with an increasing number of kinase inhibitors used clinically to treat various cancers (Klaeger et al., 2017), and the possibility of repurposing human kinase inhibitors. Various kinase inhibitors have been identified for kinetoplastids (Pena et al., 2015; Woodland et al., 2015; Amata et al., 2016; Wyllie et al., 2018) and *Plasmodium* spp. (Hallyburton et al., 2017; Mathews and Odom John, 2018), with some showing clinical promise. But what about cytokinesis kinases? Repurposing and refinement of human aurora kinase inhibitors has identified effective and selective compounds for *T. brucei* and *P. falciparum in vitro* (Ochiana et al., 2013; Patel et al., 2014). Further, PfPKG inhibitory thiazole compounds with additional activity against PfCDPK4, PfCRK5, and PfNEK1 have been identified (Penzo et al., 2019), as well as PfCDPK4 inhibitors that block exflagellation and hence, likely, transmission of *P. falciparum* (Ojo et al., 2014; Vidadala et al., 2014). TgMAPKL-1, targeted by bumped kinase inhibitors, is reported to have promising drug target potential (Sugi et al., 2013, 2015; Brown et al., 2014), TbPK50 and TbPK53 kinase inhibitors have been identified (Ma et al., 2010; Urbaniak et al., 2012) and the CK2 inhibitor, emodin, inhibits cytokinesis in *T. cruzi* (De Lima et al., 2017). Aside from protein kinases, sphingosine derivatives and inhibitors of sphingolipid biosynthetic enzymes have potential against *Giardia* (Sonda et al., 2008; Stefanic et al., 2010) and *T. brucei* (Fridberg et al., 2008; Jones et al., 2015), while repurposing of human PDE inhibitors shows promise in *T. brucei* (de Koning et al., 2012; Ochiana et al., 2015). In the future, it may also be possible to repurpose inhibitors of human Plk1 (Gutteridge et al., 2016), Hippo pathway inhibitors (Qiao et al., 2019) or enzymes such as katanin/spastin as they are developed (Pisa et al., 2019). However, currently, further work is required to refine existing cytokinesis inhibitors before they will be clinically useful.

### Conclusions

Protozoan parasites cause great morbidity and mortality in humans and animals worldwide, and are therefore of considerable medical and economic concern. They are also of great evolutionary interest, with the multiple and varied modes of cytokinesis highlighting the diversity of cell biology in non-model organisms. Indeed, across the evolutionary tree, only a relatively small proportion of organisms use a canonical actomyosin ring to divide. Increasing our knowledge of the molecular pathways that underpin such diverse biology will aid our understanding of the evolution of cytokinesis and potentially uncover novel drug discovery approaches for protozoan parasites.

## Author Contributions

TH conceived, researched, and wrote the article.

### Conflict of Interest

The author declares that the research was conducted in the absence of any commercial or financial relationships that could be construed as a potential conflict of interest.
